# Carboxyl-Functionalized Lotus Cellulose Fiber-Based Superabsorbent Hydrogel for Efficient Water and Fertilizer Management

**DOI:** 10.3390/gels12090854

**Published:** 2026-09-20

**Authors:** Jiawen Chen, Guohang Ma, Keting Chen, Tiansheng Wang, Erlei Zhang, Jianmin Li, Bao Han, Wenfeng Lu, Jichuan Zhang, Jiaheng Zhang

**Affiliations:** 1 Sauvage Laboratory for Smart Materials, Harbin Institute of Technology, Shenzhen 518055, China; 2 School of Materials Science and Engineering, Harbin Institute of Technology, Shenzhen 518055, China; 3 State Key Laboratory of Advanced Welding and Joining and Research Centre of Printed Flexible Electronics, School of Materials Science and Engineering, Harbin Institute of Technology, Shenzhen 518055, China; 4 School of Life Sciences, Jilin University, Changchun 130000, China; 5 Shenzhen Shinehigh Innovation Technology Co., Ltd., Shenzhen 518055, China

**Keywords:** cellulose hydrogel, water absorbency, fertilizer retention, agricultural waste valorization, swelling

## Abstract

To address challenges associated with the water–food nexus under climate change and population growth, a multifunctional cellulose-based superabsorbent hydrogel (L-CF/CMCAA-1ATP) was developed by incorporating carboxyl-functionalized lotus cellulose fibers (L-CF) derived from waste lotus stems via deep eutectic solvent extraction and spray drying into a carboxymethyl cellulose/acrylic acid/attapulgite hydrogel network. Under optimized conditions, the L-CF/CMCAA-1ATP hydrogel exhibited an equilibrium swelling capacity of 1979 g/g in deionized water and retained 74.37% of absorbed water after 250 min at 75 °C. Soil application significantly improved water-holding capacity (40.11% to 56.55%) and maintained 34.41% water retention after 21 days. Additionally, cumulative nitrogen, phosphorus, and potassium losses were reduced by 28.84%, 28.56%, and 42.23%, respectively, after five leaching cycles, with DFT calculations indicating that nutrient retention arises from hydrogen-bonding interactions with carboxyl, hydroxyl, and siloxane groups in the hydrogel network. Agricultural assays demonstrated enhanced maize growth, with chlorophyll content increasing by 21.56% compared with the control. Cell viability remained above 95% at all tested concentrations, confirming low cytotoxicity. These results demonstrate that the L-CF/CMCAA-1ATP superabsorbent hydrogel combines excellent water absorbency, water retention, nutrient conservation, plant growth promotion, and low in vitro cytotoxicity, highlighting its potential as a waste-valorization approach for utilizing agricultural residues and improving water and nutrient management in agriculture.

## 1. Introduction

The global water crisis is driven by population growth, economic development, water pollution, and climate change [1,2]. Agriculture, as a highly water-intensive sector, accounts for roughly 70% of global freshwater withdrawals, yet its productivity remains insufficient to meet growing food demand [3]. Since the early 21st century, global irrigated land has expanded by approximately 52%, further exacerbating regional water stress [4]. Inefficiencies and water wastage in agricultural practices significantly limit improvements in water-use efficiency [5]. Meanwhile, conventional fertilizers are highly soluble, causing severe nutrient losses and environmental problems such as water eutrophication, soil acidification, and greenhouse gas emissions [6,7,8]. Developing superabsorbent hydrogels (SAHs) with high water retention capabilities is therefore increasingly important. SAHs can improve soil hydraulic properties, reduce nutrient leaching, enhance water and fertilizer utilization efficiency, and provide a sustainable hydrogel-based strategy for mitigating agricultural water stress and environmental pollution [9,10,11].

SAHs are crosslinked hydrogel networks capable of absorbing and retaining large amounts of water while simultaneously reducing nutrient loss through controlled release, making them promising materials for improving water and fertilizer utilization efficiency in agriculture [12,13,14,15]. However, most commercial SAHs are synthesized from fossil-derived monomers such as acrylic acid and acrylamide, raising concerns regarding cost, biodegradability, and environmental sustainability [16]. Consequently, bio-based SAHs derived from natural polymers have attracted increasing attention as potential alternatives with greater renewable-resource content [17].

Polysaccharides, particularly cellulose and its derivatives, have emerged as promising biopolymers for the synthesis of bio-SAHs due to their abundance, low cost, biodegradability, and nontoxicity [18]. However, cellulose-based SAHs often suffer from limited swelling capacity, poor water-retention performance, and insufficient stability under practical agricultural conditions compared with fully synthetic SAHs [19]. To address these limitations, hybrid strategies incorporating synthetic monomers and inorganic fillers have been extensively explored. Previous studies have demonstrated that cellulose fibers, clay minerals, biochar, and other functional components can significantly improve the swelling behavior, mechanical stability, and environmental adaptability of SAHs [8,12,20,21]. Chemical modification is an effective approach to improve cellulose functionality by enriching hydrophilic and ionizable groups, such as carboxyl and hydroxyl groups, thereby enhancing water uptake, network swelling, and interactions with nutrient species [11,22]. In addition to compositional optimization, tailoring the morphology of cellulose through techniques such as spray drying can further enhance water transport, storage, and controlled-release properties by increasing particle porosity and surface area [23,24].

From the perspectives of resource utilization and biomass valorization, agricultural biomass residues and naturally abundant minerals have attracted increasing attention in the development of high-performance SAHs. Lotus stems are abundant agricultural by-products rich in cellulose and characterized by intrinsic porous structures and capillary channels, making them promising feedstocks for bio-based hydrogel materials [25,26]. Attapulgite (ATP), a low-cost layered aluminosilicate containing exchangeable cations, features a unique chain-layered structure and an abundance of surface hydroxyl groups [27]. Incorporation of ATP in hydrogel composites has been shown to improve mechanical strength, thermal stability, and swelling and adsorption properties [10,28,29]. Therefore, the combination of porous biomass-derived cellulose and ATP provides a promising strategy for constructing multifunctional cellulose-based superabsorbent hydrogels with enhanced water management and agricultural performance. Building on these complementary advantages, biomass-derived cellulose and clay minerals have recently been combined to construct multifunctional agricultural superabsorbent hydrogels [8,30,31]. However, biomass-derived fibers in reported cellulose/clay hydrogels are often used in their native or minimally processed form. The incorporation of agricultural waste-derived cellulose that is further chemically functionalized and morphologically tailored, particularly through carboxylation and spray drying, remains less explored.

Herein, a waste valorization strategy for waste lotus stems was developed. Cellulose was extracted from lotus stem residues using a deep eutectic solvent (DES, citric acid/choline chloride) system, followed by spray drying to obtain lotus cellulose fibers (L-CF). The resulting L-CF was incorporated into a carboxymethyl cellulose/acrylic acid/attapulgite (CMC/AA/ATP) system to develop a multifunctional SAH via ammonium persulfate (APS)-initiated graft copolymerization. The chemical structure, crystallinity, thermal stability, and morphology of the hydrogel material were characterized using Fourier transform infrared spectroscopy (FTIR), X-ray diffraction (XRD), thermogravimetric analysis (TGA), and scanning electron microscopy with energy-dispersive spectroscopy (SEM-EDS), respectively. Water retention capacity under different temperatures, along with soil water-holding and water retention performance at varying application rates, were also evaluated. In addition, density functional theory (DFT) calculations were employed to elucidate fertilizer–hydrogel interactions, while maize cultivation and cytotoxicity assays were conducted to evaluate its agronomic performance and cytotoxicity, respectively. By converting waste-derived cellulose into a carboxyl-functionalized and morphologically tailored reinforcement and integrating it with ATP, this study provides a strategy for constructing cellulose/clay-based SAHs with enhanced water retention, nutrient retention, and crop growth performance, with potential for agricultural water and fertilizer management.

## 2. Results and Discussion

### 2.1. Mechanism of L-Cf/cmcaa-1atp

**Figure 1:**
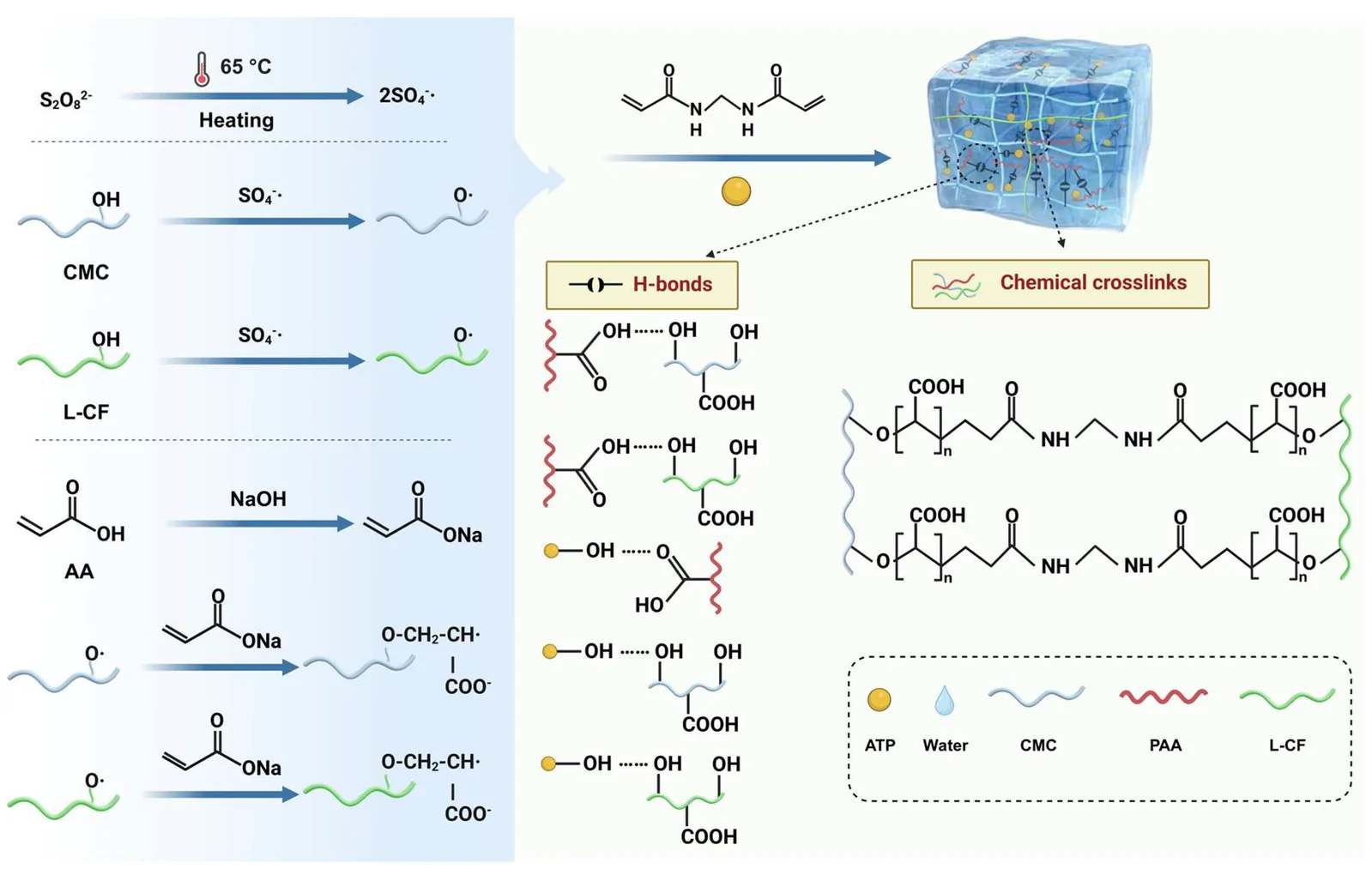
The reaction mechanism for synthesis of L-CF/CMCAA-1ATP (Created in Biorender. Jiawen Chen. (2026) https://app.biorender.com/illustrations/6a856ae37ffb440c243ac806, accessed on 14 September 2026).

The schematic illustration of the fabrication process for L-CF/CMCAA-1ATP is presented in Figure 1. During hydrogel synthesis, graft-copolymerization and crosslinking reactions occur among AA, CMC, L-CF, N,N′-methylenebisacrylamide (MBA, as the crosslinker), and APS, as the initiator, leading to the formation of the superabsorbent hydrogel network. L-CF, derived from waste lotus stems via DES extraction and spray drying, contains abundant hydroxyl and carboxyl functional groups, enabling it to participate in both hydrogen bonding and chemical crosslinking with the PAA/CMC chains within the hydrogel network. Concurrently, ATP is incorporated as an inorganic reinforcing filler and physical crosslinking component within the hydrogel network to yield the L-CF/CMCAA-1ATP. The specific reaction mechanism is proposed as follows: under thermal treatment, APS decomposes to generate sulfate radicals, which indicates the production of active radicals such as R-O· in the CMC and L-CF structure and further leads to a polymerisation reaction with AA [32]. During the polymerization process, the terminal vinyl groups of MBA react with the propagating PAA chains and the CMC and L-CF backbones, effectively functioning as bridge-linking agents to establish a robust three-dimensional chemically cross-linked hydrogel network [18]. Hydrogen bonding interactions form between the –OH groups of CMC and L-CF and the –COO^-^ groups of PAA [33,34]. In addition, ATP contributes to hydrogel network formation through hydrogen bonding between its surface silanol (Si–OH) groups and the carboxyl groups of PAA and L-CF, as well as the hydroxyl groups of CMC and L-CF. ATP also acts as a physical crosslinking node embedded within the hydrogel network [35,36]. The above interactions collectively form a physical–chemical cross-linked hydrogel network.

### 2.2. Characterization of L-CF

**Figure 2:**
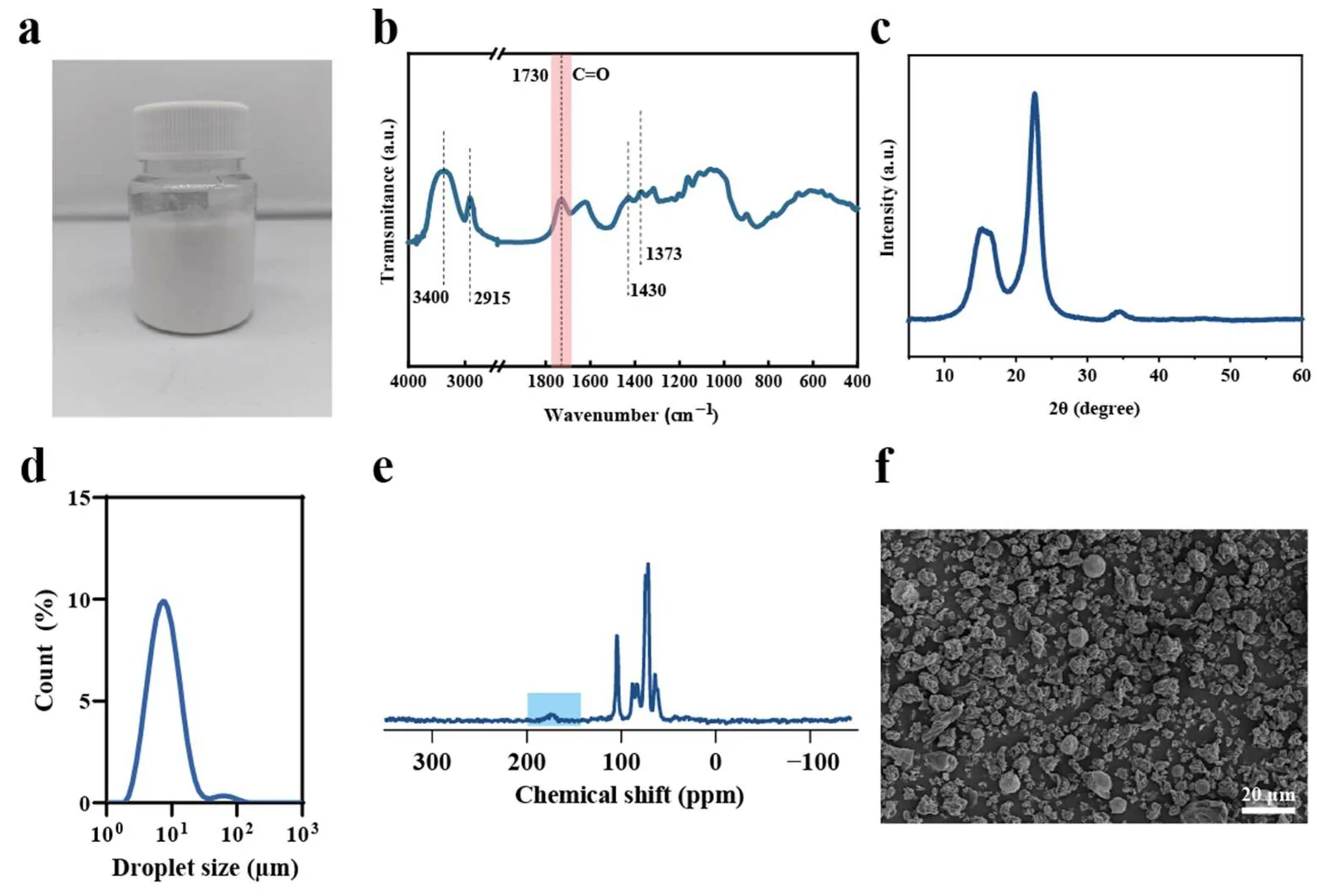
Photograph (**a**), FTIR spectrum (**b**), XRD pattern (**c**), particle size distribution (**d**), ^13^C solid-state nuclear magnetic resonance (ssNMR) spectrum (**e**) and SEM (**f**) of L-CF.

Lotus stems were subjected to DES extraction followed by bleaching, yielding a white powdered L-CF (Figure 2a). The DES extraction process achieved a cellulose yield of 33% and the extracted L-CF exhibited a cellulose content of 99 wt%. The FTIR spectrum of L-CF (Figure 2b) exhibited the characteristic peaks of cellulose: O–H stretching at 3400 cm^−1^, C–H stretching at 2915 cm^−1^, H–C–H and O–C–H in-plane bending at 1430 cm^−1^, and C–H deformation at 1373 cm^−1^ [37]. The C=O stretching band observed at 1730 cm^−1^ was attributed to the carboxyl groups of citric acid, indicating their successful incorporation into the L-CF during DES treatment [38]. The crystal structure of L-CF was examined using XRD (Figure 2c). L-CF displayed three characteristic XRD peaks at 2θ ≈ 16.2°, 22.7°, and 34.5°, corresponding to the (110), (200), and (004) crystal planes of cellulose I_β_, respectively [39]. The particle size distribution showed a peak at 7.59 µm (Figure 2d). The appearance of additional signals at 165–185 ppm, assigned to the carbonyl carbons of ester and carboxyl groups derived from citric acid, confirmed the successful carboxylation of L-CF (Figure 2e) [40]. Conductometric titration quantitatively determined the carboxyl-group content of L-CF to be 0.87 mmol/g, corresponding to a degree of substitution of approximately 0.14. This quantitative result, together with the FTIR and ssNMR data, confirms the successful introduction of carboxyl functionality into the cellulose backbone. The introduced carboxyl groups provide additional ionizable and hydrophilic sites, which may contribute to the subsequent swelling behavior of the hydrogel and its interactions with nutrient species. The spray-dried L-CF appeared as irregular, wrinkled spherical particles with a rough surface (Figure 2f), which may provide a high surface area and enhance adsorption capacity [23].

### 2.3. Characterization of Polymers and Sahs

The gel fractions of CMCAA, CMCAA-1ATP, and L-CF/CMCAA-1ATP were 82.23 ± 2.50%, 87.03 ± 1.27%, and 95.25 ± 1.19%, respectively, corresponding to sol fractions of 17.77 ± 2.50%, 12.97 ± 1.27%, and 4.75 ± 1.19%. The high gel fractions confirmed the successful formation of insoluble crosslinked networks. Moreover, the progressive increase in gel fraction after the incorporation of ATP and L-CF suggests that these components promoted the formation of a more complete and stable hydrogel network.

**Figure 3:**
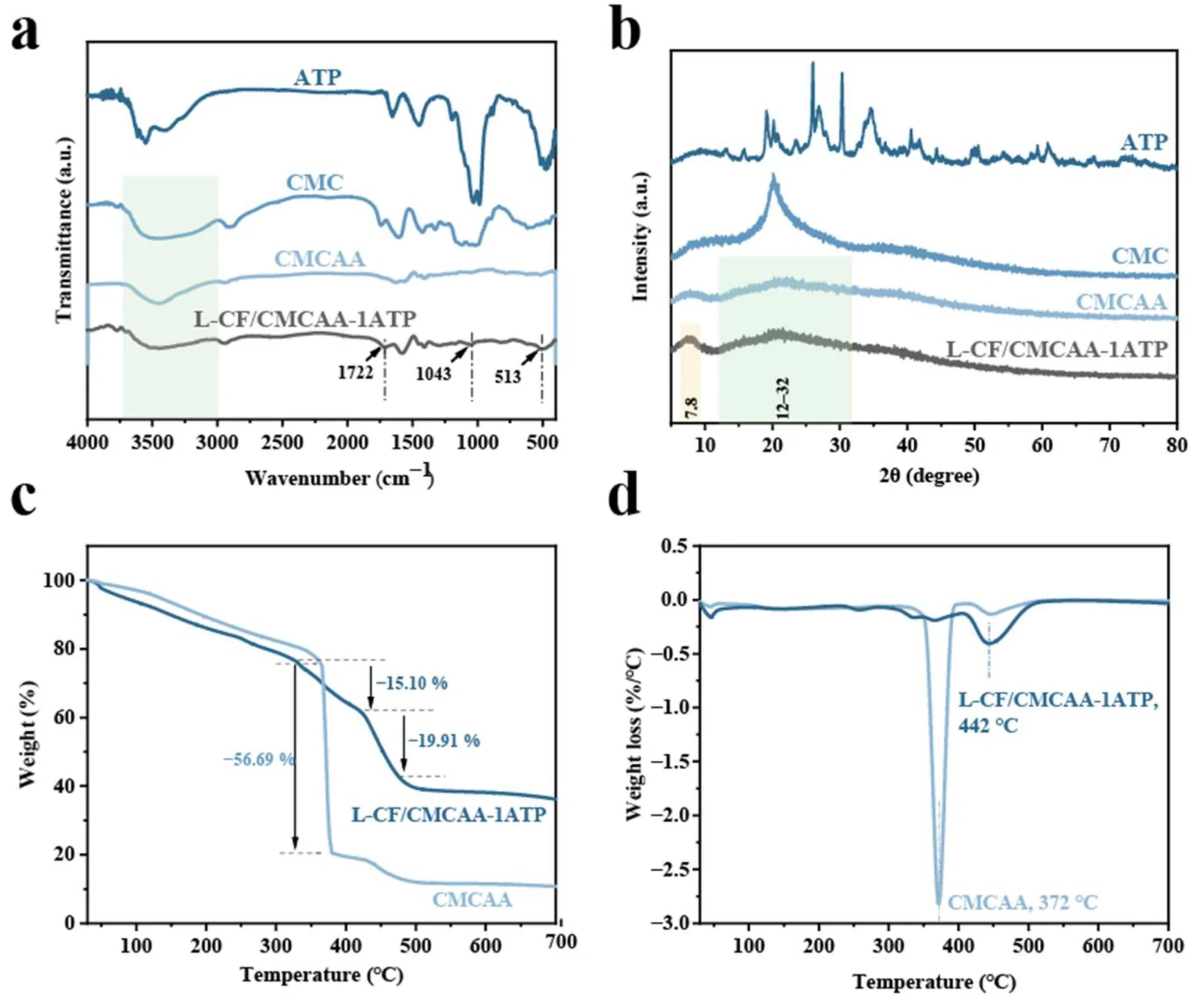
FTIR spectra (**a**) and XRD patterns (**b**) of ATP, CMC and SAHs; TGA (**c**) and derivative thermogravimetric (DTG) (**d**) curves of CMCAA and L-CF/CMCAA-1ATP.

The chemical structure of the SAHs was analyzed using FTIR spectroscopy (Figure 3a). ATP exhibited characteristic peaks: Si–O–Si asymmetric stretching vibrations at 1030 cm^−1^, Si–OH stretching near 990 cm^−1^ [41], while bending vibrations of Si–O–Al and Si–O–Si were observed at 518 cm^−1^ and 476 cm^−1^, respectively [42]. CMC showed a broad O–H stretching band at 3000–3750 cm^−1^ and –COO^−^ asymmetric/symmetric stretching at 1605 and 1422 cm^−1^ [15]. In the CMCAA sample (containing 5 wt% CMC), the O–H band shifted to a lower wavenumber (3450 cm^−1^), indicating the formation of a stronger and more extensive hydrogel network during the grafting process [17].

For L-CF/CMCAA-1ATP, the O–H band became broader and weaker, reflecting enhanced hydrogen bonding among L-CF, ATP, and the polymer hydrogel network [43]. The band at 1722 cm^−1^ was assigned to C=O stretching of carboxyl-containing groups and was consistent with the presence of carboxyl-functionalized L-CF in the hydrogel network [8]. New peaks at 1043 and 513 cm^−1^ correspond to Si–O–Si stretching and Si–O–Al bending, supporting ATP incorporation into the composite hydrogel [44].

The crystalline structures of the raw materials (ATP, CMC) and the synthesized hydrogels (CMCAA, L-CF/CMCAA-1ATP) were further characterized using XRD (Figure 3b). CMC exhibited a broad diffraction peak at 2θ ≈ 19.9°, consistent with the semicrystalline structure commonly reported for CMC and cellulose II-like domains [45]. ATP exhibited several sharp diffraction peaks at 2θ values of 19.1°, 20.2°, 26.0°, and 30.3°, reflecting its highly crystalline mineral structure [35]. L-CF/CMCAA-1ATP was markedly weakened, indicating disruption of ordered cellulose domains and increased amorphous character after graft copolymerization [21]. L-CF/CMCAA-1ATP retained a diffraction peak at 2θ ≈ 7.8°, assigned to the (110) plane of ATP, indicating that part of the ATP crystalline structure was preserved in the composite hydrogel [46].

The TGA and DTG curves of CMCAA and L-CF/CMCAA-1ATP are presented in Figure 3c,d, respectively. Compared with CMCAA, L-CF/CMCAA-1ATP exhibited a broader decomposition range, a higher residual mass, and a shift in the dominant DTG peak from 372 to 442 °C. These results indicate that the incorporation of L-CF and ATP improved the thermal stability of the hydrogel network.

**Figure 4:**
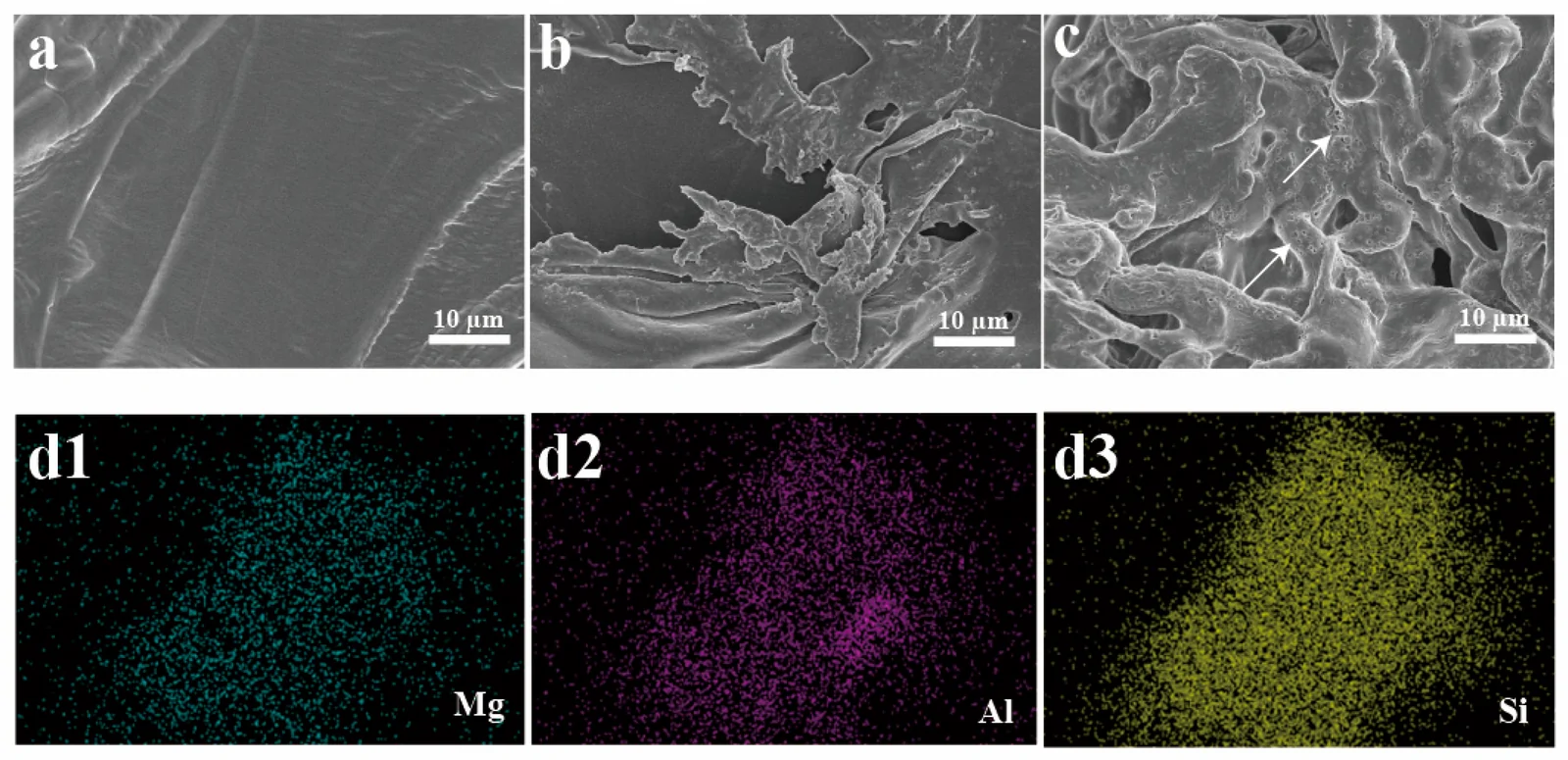
SEM images of CMC (**a**), CMCAA (**b**) and L-CF/CMCAA-1ATP (**c**), and spectra of EDS point measurements for L-CF/CMCAA-1ATP (**d1**–**d3**).

The morphologies of CMC, CMCAA, and L-CF/CMCAA-1ATP after swelling equilibrium followed by freeze-drying were characterized by SEM (Figure 4). The surface of CMC was smooth and dense. After polymerization, CMCAA showed a looser hydrogel structure with partially interconnected pores. In comparison, L-CF/CMCAA-1ATP displayed a well-developed porous hydrogel network. Such structural features may contribute to water transport and storage within the hydrogel matrix [10]. EDS further confirmed the presence of Mg, Al, and Si in L-CF/CMCAA-1ATP (Figure 4d1–d3, Figure S1), supporting the successful incorporation of ATP into the hydrogel network.

### 2.4. Effects of Synthesis Parameters and L-Cf Incorporation on the Swelling Behavior of SAHs

The synthesis conditions strongly influence the network structure and swelling performance of SAHs [47]. Therefore, the effects of the ND, ATP content, and CMC content (all expressed as weight percentages relative to AA) on the swelling behavior of the hydrogels were systematically investigated.

#### 2.4.1. Effects of Neutralization Degree of AA

**Figure 5:**
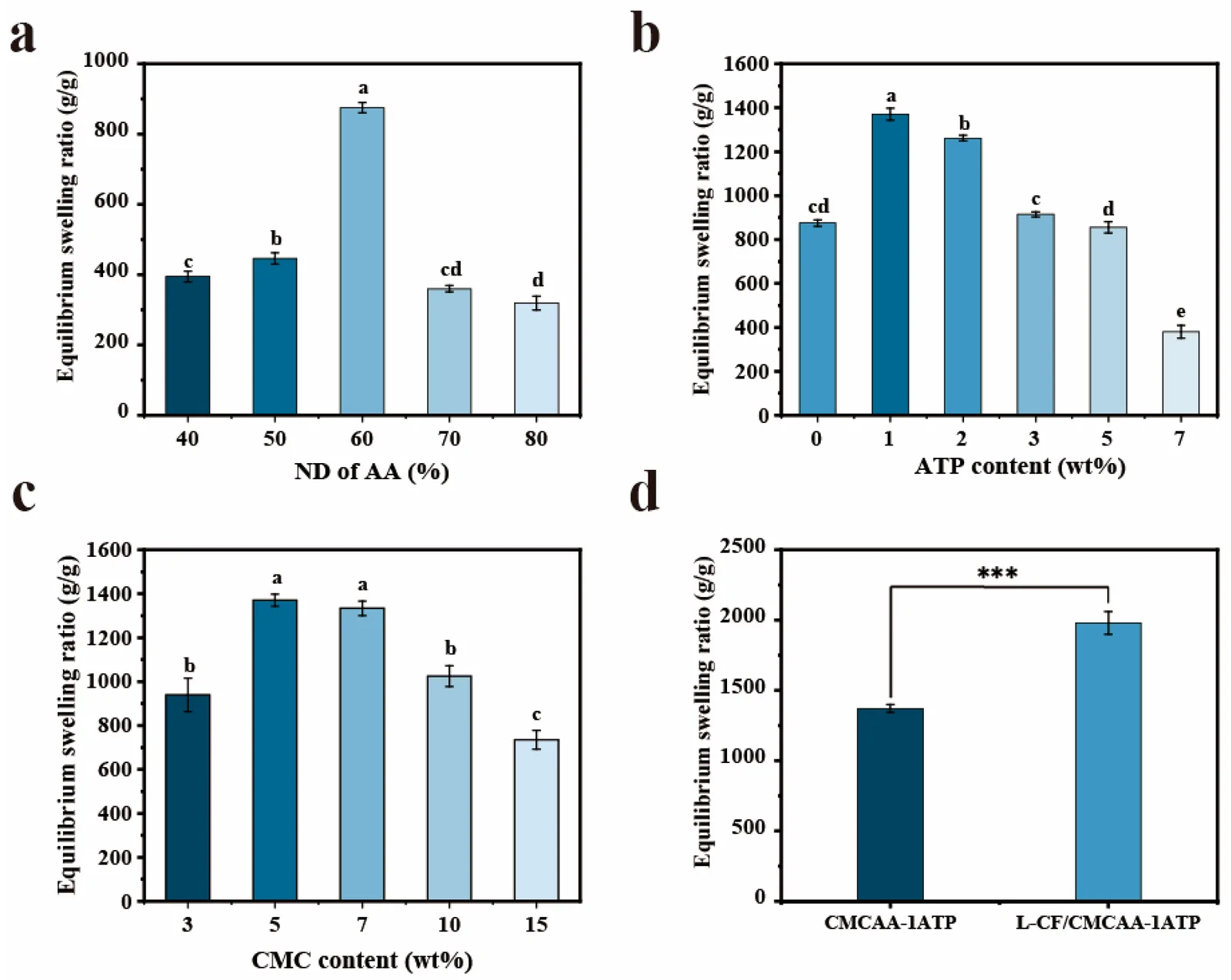
Effects of neutralization degree (ND) of AA (**a**), ATP content (**b**) and CMC content (**c**) on the equilibrium swelling ratio of SAHs (all variables are relative to AA (wt%)). (**d**) Comparison of the equilibrium swelling ratio between CMCAA-1ATP and L-CF/CMCAA-1ATP. Different lowercase letters (a–e) indicate significant differences (*p* < 0.05) among treatments. *** indicates *p* < 0.001. Error bars represent mean ± standard deviation (SD).

As the ND of AA increased from 40% to 80%, the equilibrium swelling ratio (Q_e_) in deionized water showed a non-linear trend, increasing from 395 g/g at 40% to 446 g/g at 50%, reaching a maximum of 875 g/g at 60% (*p* < 0.0001), and then decreasing to 360 g/g and 320 g/g at 70% and 80%, respectively (Figure 5a).

At low ND, the limited ionization of carboxyl groups results in weak electrostatic repulsion and low osmotic pressure within the network, thereby restricting water uptake [48]. Increasing ND from 40% to 60% elevates the density of carboxylate groups and Na^+^ ions, enhancing osmotic pressure and promoting water absorption. Beyond 60%, electrostatic screening and ionic equilibrium between COO^−^ groups and Na^+^ ions dominate, limiting hydrogel network expansion and reducing the swelling capacity [11,49].

#### 2.4.2. Effects of ATP Content

As the mass ratio of ATP to AA increased from 0% (CMCAA) to 1% (CMCAA-1ATP), Q_e_ significantly rose from 875 g/g to 1371 g/g (*p* < 0.0001), indicating that a small amount of ATP markedly enhances water absorption (Figure 5b). This enhancement is likely attributed to the physical cross-linking effect of ATP, which facilitates the construction of a stable and swellable hydrogel network [42]. When the ATP content was further increased to 7%, the Q_e_ decreased sharply to 381 g/g (*p* < 0.0001). This decline is likely due to excessive hydrogel network densification [15]. This trend is consistent with previous reports [10].

#### 2.4.3. Effects of CMC Content

Figure 5c shows the effect of various CMC contents on the water absorbency of the SAHs. The Q_e_ increased from 940 g/g at 3 wt% to a peak value of 1371 g/g at 5 wt% (*p* < 0.0001), and then decreased progressively to 735 g/g as the CMC content increased to 15 wt% (*p* < 0.0001). The initial increase from 3 to 5 wt% is attributed to the higher density of hydrophilic groups introduced by CMC, which promotes hydrogen bonding and facilitates water penetration into the hydrogel network [50]. However, when the CMC content exceeds 5 wt%, the increased viscosity of the reaction system reduces monomer mobility and limits radical collisions, leading to less efficient polymer formation. As a result, the hydrogel network becomes less effectively expanded during swelling, causing a decline in water absorption [10].

#### 2.4.4. Effects of L-Cf Incorporation

Based on the optimized formulation of CMCAA-1ATP, the incorporation of 2 wt% L-CF increased Q_e_ from 1371 to 1979 g/g, corresponding to an improvement of approximately 44% (*p* < 0.0001) (Figure 5d). This enhancement of hydrogel was attributed to the abundant hydroxyl and carboxyl groups and the wrinkled morphology of L-CF [16].

### 2.5. Water Absorption and Retention Properties of SAHs

#### 2.5.1. Swelling Behavior of SAHs

**Figure 6:**
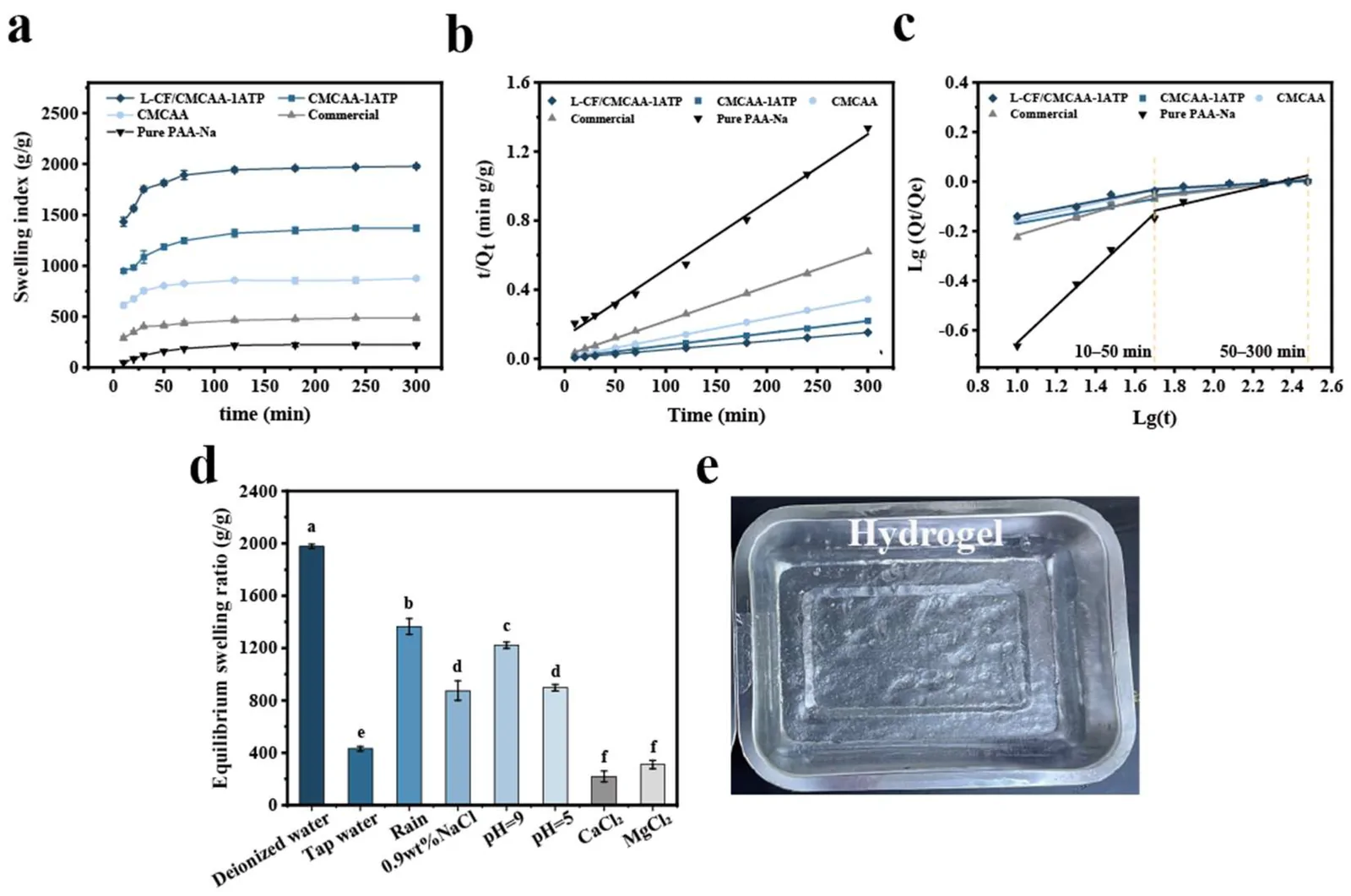
(**a**) The swelling degree curves of SAHs over time in deionized water; (**b**) pseudo-second-order kinetic fitting of SAHs; (**c**) Lg(Q_t_/Q_e_)-Lg(t) curves of SAHs; (**d**) The equilibrium swelling ratio of L-CF/CMCAA-1ATP in various environments; (**e**) The appearance of L-CF/CMCAA-1ATP before and after the absorption of deionized water. Different lowercase letters (a–f) indicate significant differences (*p* < 0.05) in equilibrium swelling ratio of the L-CF/CMCAA-1ATP in various environments. Error bars represent mean ± SD.

Figure 6a illustrates the swelling kinetics of the commercial hydrogel, the pure sodium polyacrylate hydrogel sample (pure PAA-Na), and the prepared composite SAHs in deionized water. All hydrogel samples showed a rapid swelling stage within the first 50 min, followed by a slower swelling process until equilibrium was attained at approximately 120 min. The equilibrium swelling capacities followed the order: L-CF/CMCAA-1ATP > CMCAA-1ATP > CMCAA > Commercial hydrogel > Pure PAA-Na. Notably, the equilibrium swelling index of L-CF/CMCAA-1ATP reached approximately 1970 g/g, which was 1.4-fold and 2.3-fold higher than those of CMCAA-1ATP and CMCAA, respectively. The enhanced swelling behavior can be attributed to the combined contributions of abundant hydrophilic and ionizable carboxyl groups introduced by both CMC and L-CF and the network-expanding and pore-generating role of ATP, which together facilitate water penetration and retention within the hydrogel network.

Kinetic analysis was performed using the pseudo-second-order and Ritger–Peppas models. As shown in Figure 6b, the experimental data fit excellently with the pseudo-second-order kinetic model (R^2^ > 0.99 for all samples, Table 1). L-CF/CMCAA-1ATP showed the highest calculated equilibrium swelling capacity (Q_e_, cal = 2009 g/g) and initial swelling rate (K_is_ = 427.35 g/g·min). The improved kinetic parameters further demonstrate that L-CF facilitates rapid water diffusion within the hydrogel matrix and enhances equilibrium water uptake [51].

In the Ritger-Peppas model, the diffusion exponent (n) is employed to identify the liquid diffusion mechanism. According to the kinetic parameters in Table 1 and Figure 6c, during the initial swelling stage (10–50 min), Pure PAA-Na exhibited non-Fickian transport (n = 0.7462), whereas L-CF/CMCAA-1ATP, CMCAA-1ATP, CMCAA, and the commercial hydrogel showed Fickian diffusion behavior with n < 0.45. This indicates that the incorporation of CMC, ATP, and L-CF enhanced the effective cross-linking and stability of the hydrogel network, allowing water molecules to predominantly penetrate the hydrogel matrix via physical diffusion [21]. During the later stage (50–300 min), n decreased to below 0.2, indicating an approach to hydrogel swelling equilibrium, with the hydrogel network’s elastic resistance limiting further water absorption [52]. The observed decrease in n values over time is consistent with previous studies on cellulose-based SAH swelling behavior [53].

L-CF/CMCAA-1ATP was further evaluated under different environmental conditions (Figure 6d). Q_e_ was highest in deionized water (1977 g/g), followed by rainwater (1364 g/g), pH 9 solution (1222 g/g), pH 5 solution (898 g/g), 0.9 wt% NaCl solution (875 g/g), tap water (430 g/g), MgCl_2_ solution (330 g/g), and CaCl_2_ solution (219 g/g). The markedly lower swelling ratios observed in ionic media were mainly attributed to ionic screening, whereby dissolved cations partially shield the electrostatic repulsion between negatively charged groups within the hydrogel network, thereby reducing the osmotic pressure difference and restricting network expansion and water uptake [24]. As shown in Figure 6e, L-CF/CMCAA-1ATP formed a highly swollen hydrogel after water absorption, visually demonstrating its excellent swelling capability.

#### 2.5.2. Water Retention Property of SAHs in Deionized Water

**Figure 7:**
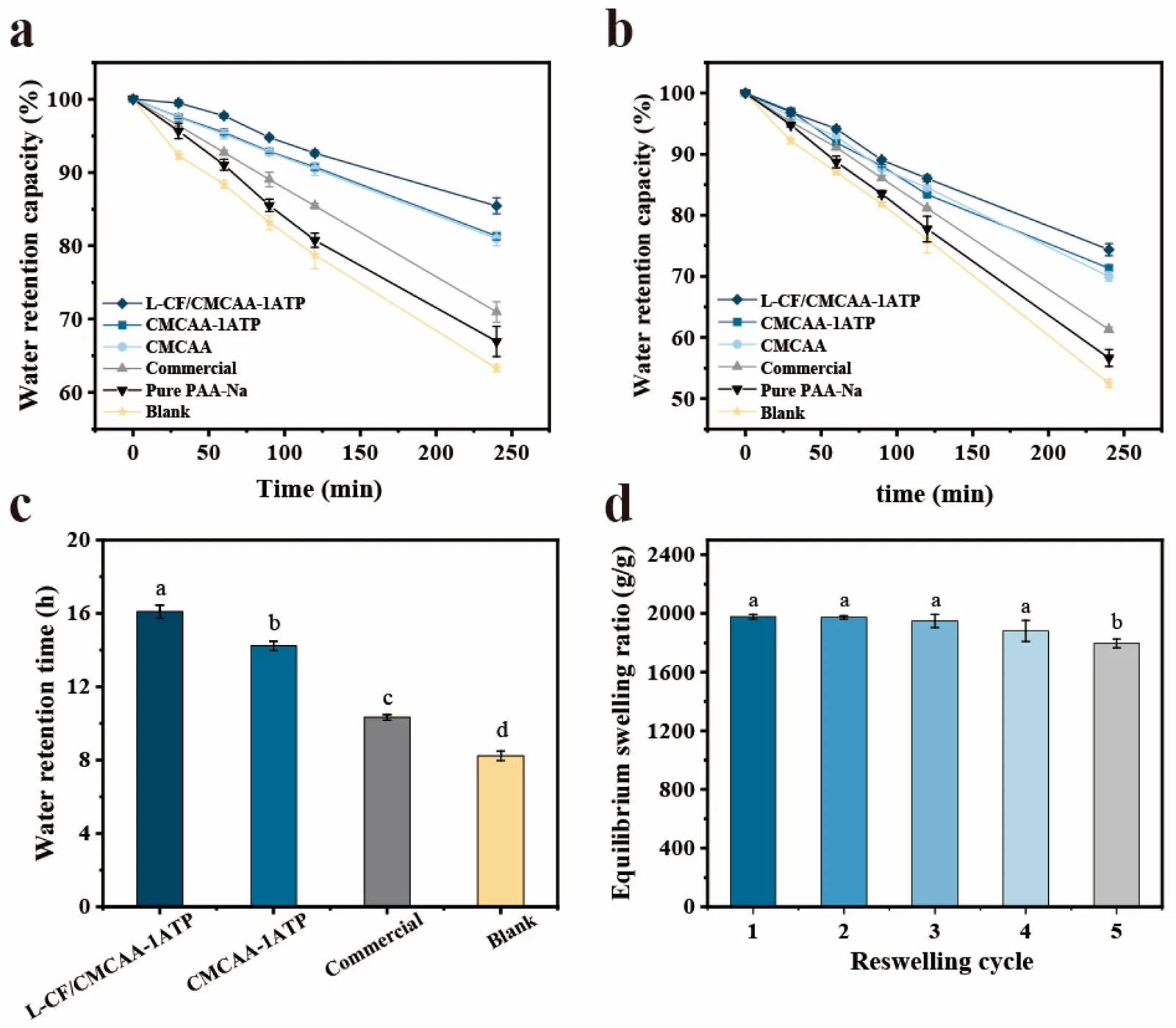
Water retention of the SAHs at 45 °C (**a**) and 75 °C (**b**); (**c**) Water retention time of the SAHs at 75 °C; (**d**) Reswelling performance of L-CF/CMCAA-1ATP over five cycles. Different lowercase letters (a–d) indicate significant differences (*p* < 0.05) in water retention time of SAHs and reswelling capacities of L-CF/CMCAA-1ATP among 5 cycles. Error bars represent mean ± SD.

The water retention performance of SAHs at elevated temperatures is critical for their practical application, particularly in arid and high-temperature agricultural environments. Therefore, the water retention behaviors of the commercial and prepared hydrogels at swelling equilibrium were systematically evaluated at 45 °C and 75 °C (Figure 7a,b). The 45 °C condition was selected to represent agriculturally relevant high-temperature conditions, as surface soil temperatures in arid and semi-arid regions can reach approximately 45–50 °C during summer [54,55]. In contrast, 75 °C was used as an extreme thermal condition to further assess the structural stability and water-retention performance of the hydrogels under severe heat stress [55]. At 250 min, L-CF/CMCAA-1ATP hydrogel exhibited the highest water retention at both temperatures, retaining 85.44% and 74.37% of absorbed water at 45 °C and 75 °C, respectively, demonstrating its superior water retention capability. As the temperature increased to 75 °C, all hydrogel samples showed a more rapid decline in water retention due to enhanced molecular motion and accelerated water evaporation at higher temperatures. Further comparison at 75 °C showed that the effective water retention times of L-CF/CMCAA-1ATP, CMCAA-1ATP, the commercial hydrogel, and the blank sample were 16.1, 14.2, 10.3, and 8.2 h, respectively (Figure 7c). The water retention time of L-CF/CMCAA-1ATP was therefore extended by 1.9 h (*p* < 0.001), 3.9 h (*p* < 0.0001) and 6.0 h (*p* < 0.0001) compared with CMCAA-1ATP, the commercial and blank samples, respectively. This enhanced water-retention performance was attributed to the synergistic effects of the rigid inorganic framework of ATP and the abundant hydrophilic groups provided by L-CF, CMC, and AA, which strengthened water–polymer interactions and reduced water loss from the hydrogel network [8]. The retention performance observed at 75 °C further indicates that the hydrogel network remained functional under extreme thermal conditions.

#### 2.5.3. Reswelling Capability of SAHs in Deionized Water

Figure 7d shows the reswelling performance of L-CF/CMCAA-1ATP during repeated swelling–deswelling cycles in deionized water. During the first three cycles, the water absorbency of L-CF/CMCAA-1ATP was 1977, 1973, and 1948 g/g, respectively. No significant decrease in water-absorption capacity was observed during the first four reswelling cycles, providing functional evidence that the hydrogel retained its network integrity during repeated swelling–drying processes. After five cycles, the water absorbency decreased to 1797 g/g, which still retained 90.9% of its initial swelling capacity. The excellent cycling stability can be attributed to the combined contributions of the hydrogel components to the stable network structure [56].

### 2.6. Water Retention Capability of SAHs in Soil

**Figure 8:**
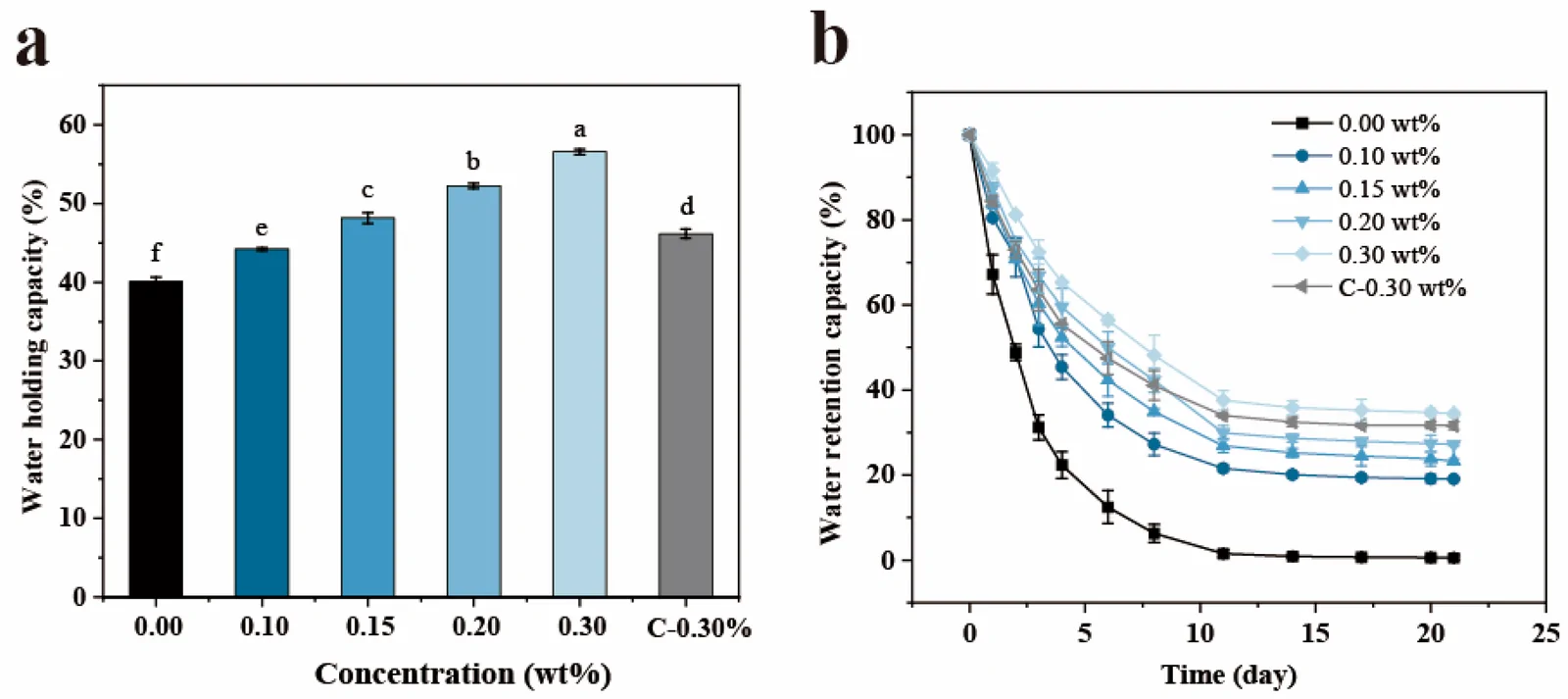
Water holding (**a**) and water retention capacity (**b**) of soil treated with different concentrations of L-CF/CMCAA-1ATP and the commercial SAH (C-0.30 wt%). Different lowercase letters (a–f) indicate significant differences (*p* < 0.05) in water-holding capacity of L-CF/CMCAA-1ATP at different concentrations and a commercial hydrogel (C-0.30 wt%). Error bars represent mean ± SD.

To evaluate the practical applicability of the SAHs in soil systems, the effects of different concentrations (0–0.30 wt%) of L-CF/CMCAA-1ATP and a commercial hydrogel on soil water holding capacity (W_h_) and water retention capacity (W_r_) were investigated (Figure 8a,b).

The blank soil (0 wt%) exhibited a W_h_ value of 40.11%. After the incorporation of L-CF/CMCAA-1ATP, the W_h_ increased significantly, reaching 56.55% at an application rate of 0.30 wt% (Figure 8a). Under the same dosage, the soil treated with the commercial hydrogel showed a lower W_h_ value of 46.14%, indicating that L-CF/CMCAA-1ATP provided superior water-holding performance.

The soil water retention behavior at different SAH addition levels is presented in Figure 8b. Throughout the 21-day test period, soils containing SAHs consistently retained more water than the blank group. In addition, the water retention capacity gradually increased with increasing L-CF/CMCAA-1ATP content. After 21 days, the blank soil was nearly completely dehydrated (W_r_ = 0.54%), whereas the soil containing 0.30 wt% L-CF/CMCAA-1ATP still maintained a W_r_ value of 34.41%. This value was 2.79% higher than that of the soil treated with the same amount of the commercial hydrogel.

The enhanced soil water-holding and water retention performance highlights the potential of L-CF/CMCAA-1ATP as an efficient water-retaining hydrogel for agricultural applications.

### 2.7. Fertilizer Retention Performance of Sahs in Soil

**Figure 9:**
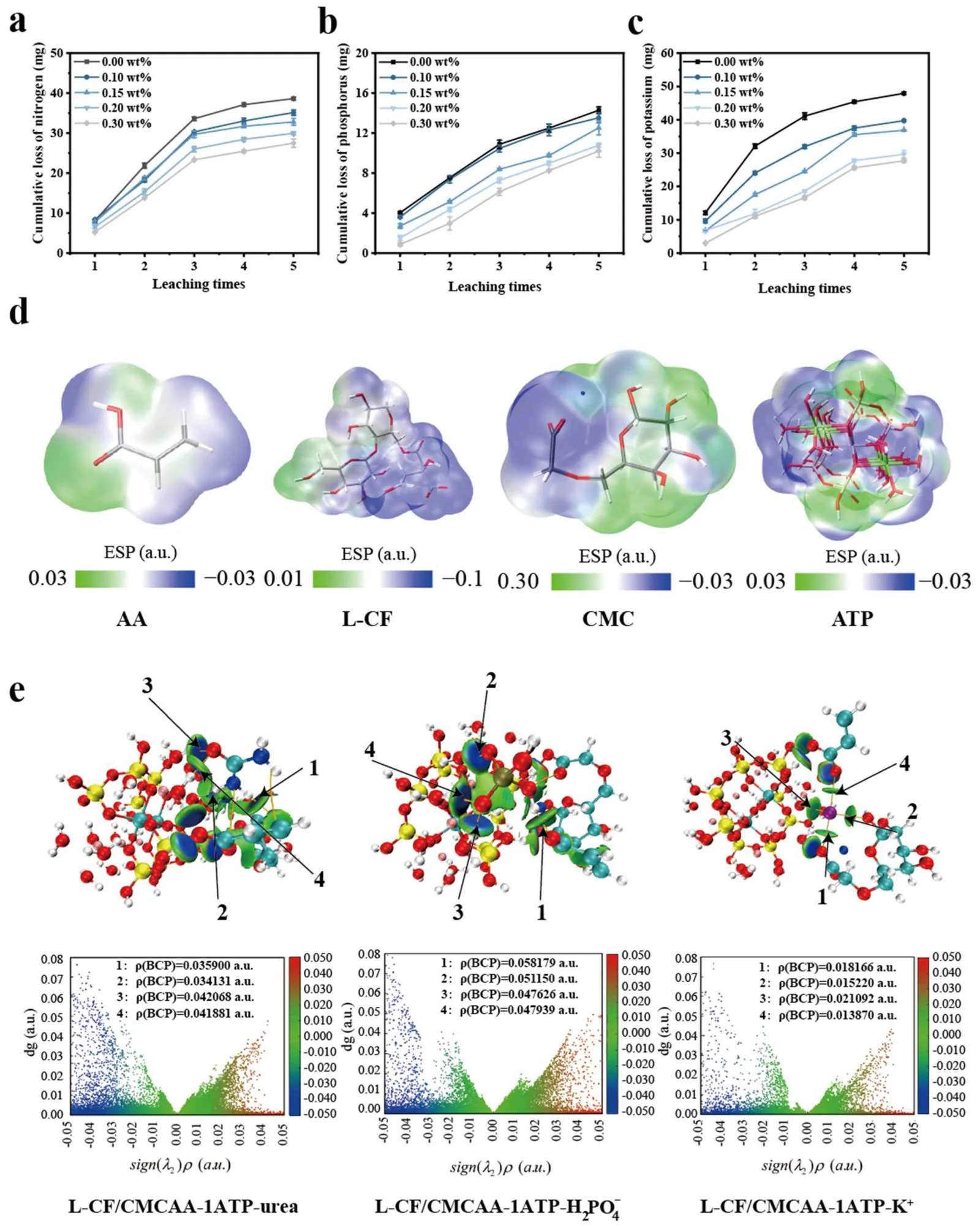
Cumulative loss of nitrogen (**a**), phosphorus (**b**) and potassium (**c**) from soil over five leaching times treated with L-CF/CMCAA-1ATP at different application rates; (**d**) Electrostatic potential (ESP) maps of AA, L-CF, CMC and ATP; (**e**) Independent gradient model based on Hirshfeld partition (IGMH) analysis of interactions between the L-CF/CMCAA-1ATP and urea, H_2_PO_4_^−^, and K^+^. Error bars represent mean ± SD.

Water movement driven by irrigation and rainfall is a major factor in the leaching of soluble nutrients from soil, reducing fertilizer-use efficiency and potentially transporting these nutrients into surrounding water bodies, where they can adversely affect aquatic ecosystems [57]. To assess the nutrient-retention capacity of the SAHs, a simulated leaching experiment was conducted to examine how different dosages (0–0.30 wt%) of L-CF/CMCAA-1ATP influenced cumulative nitrogen, phosphorus, and potassium losses in soil (Figure 9).

Compared with the control group (0 wt%), all soils amended with SAHs showed markedly reduced nitrogen leaching, with mitigation increasing alongside SAH dosage (Figure 9a). The initial nitrogen input was approximately 51.3 mg per column, derived from 110 mg of urea. After five leaching cycles, the cumulative nitrogen loss in the control group was 38.63 mg, whereas those in the 0.10, 0.15, 0.20, and 0.30 wt% SAH groups were 35.12, 32.79, 29.95, and 27.49 mg, respectively. Cumulative nitrogen losses decreased by 9.07%, 15.11%, 22.47%, and 28.84% for 0.10, 0.15, 0.20, and 0.30 wt% SAH, respectively.

Phosphorus, due to its strong adsorption and immobilization in soil, exhibited slower leaching, yet environmental risks of eutrophication remain. The initial phosphorus input was approximately 45.5 mg per column, derived from 200 mg of KH_2_PO_4_. After five leaching cycles, cumulative phosphorus losses were 14.28, 13.53, 12.53, 10.78, and 10.20 mg for the control and the 0.10, 0.15, 0.20, and 0.30 wt% SAH groups, respectively. At day 25, cumulative phosphorus losses were reduced by 5.29%, 12.26%, 24.53%, and 28.56% with increasing SAH dosages (Figure 9b). Potassium, being highly soluble, showed rapid leaching, reaching 82.65% in the control group by day 25. The initial potassium input was approximately 58.0 mg per column, derived from 200 mg of KH_2_PO_4_. After five leaching cycles, cumulative potassium losses were 47.94, 39.73, 36.85, 29.65, and 27.69 mg for the control, 0.10, 0.15, 0.20, and 0.30 wt% SAH groups, respectively. Incorporation of L-CF/CMCAA-1ATP substantially inhibited this loss, reducing cumulative potassium leaching to 68.50%, 63.54%, 51.13%, and 47.74% as SAH dosage increased (Figure 9c).

Overall, L-CF/CMCAA-1ATP effectively suppressed the leaching of nitrogen, phosphorus, and potassium. This performance is attributed to its porous three-dimensional hydrogel network, which forms upon water absorption and swelling within the soil matrix. The hydrogel network acts as a physical barrier and maintains a high internal ionic concentration [11], slowing water infiltration and providing a buffer zone that retains nutrients for plant availability.

Nitrogen and phosphorus were introduced as urea and KH_2_PO_4_, respectively, which exist in soil solution as urea, K^+^, and H_2_PO_4_^−^ ions and were used in the DFT analysis. L-CF/CMCAA-1ATP was selected as a representative model of the hydrogel network for subsequent DFT analysis.

ESP analysis identified the oxygen-containing groups of AA, L-CF, CMC, and ATP as the primary interaction sites for nutrient species within the hydrogel network (Figure 9d). The negatively charged oxygen atoms in the carboxyl, hydroxyl, and silanol groups favored interactions with K^+^ through ion–dipole/ion interactions, whereas the positively charged regions promoted electrostatic interactions with H_2_PO_4_^−^. In addition, the abundant –OH, –COOH/–COO^−^, and Si–O groups provided multiple hydrogen-bonding sites for urea, facilitating its interaction with the hydrogel network [11].

IGMH and atoms-in-molecules (AIM) analyses further confirmed the noncovalent interactions between L-CF/CMCAA-1ATP and the nutrient species (Figure 9e) [58]. AIM analysis provided quantitative information on their interaction strength through the electron density at the bond critical point (ρBCP). Hydrogen bonds with a ρBCP value higher than 0.042 are generally classified as strong hydrogen bonds. Among them, H_2_PO_4_^−^ exhibited the highest electron density at the bond critical point (ρBCP = 0.058 a.u.) and the greatest number of strong hydrogen bonds (urea: 2, H_2_PO_4_^−^: 4, K^+^: 0), indicating the strongest interaction with the hydrogel network [59]. Consistently, the IGMH isosurfaces revealed more extensive interaction regions for H_2_PO_4_^−^ than for urea and K^+^, suggesting the formation of a more stable multisite interaction network within the hydrogel matrix. In contrast, K^+^ showed the weakest interaction with the hydrogel matrix, which is qualitatively consistent with its relatively higher mobility observed during the soil leaching experiment. However, these DFT calculations represent simplified molecular-level interactions and cannot fully reproduce the complexity of nutrient retention in real soil. In practice, nutrient retention is also influenced by hydrogel swelling and physical entrapment, water transport, ion exchange, and interactions with soil components. Therefore, the DFT results should be regarded as complementary molecular-level evidence rather than a complete explanation of the soil nutrient-retention behavior.

### 2.8. Evaluation of SAHs in Promoting Maize Seedling Growth

**Figure 10:**
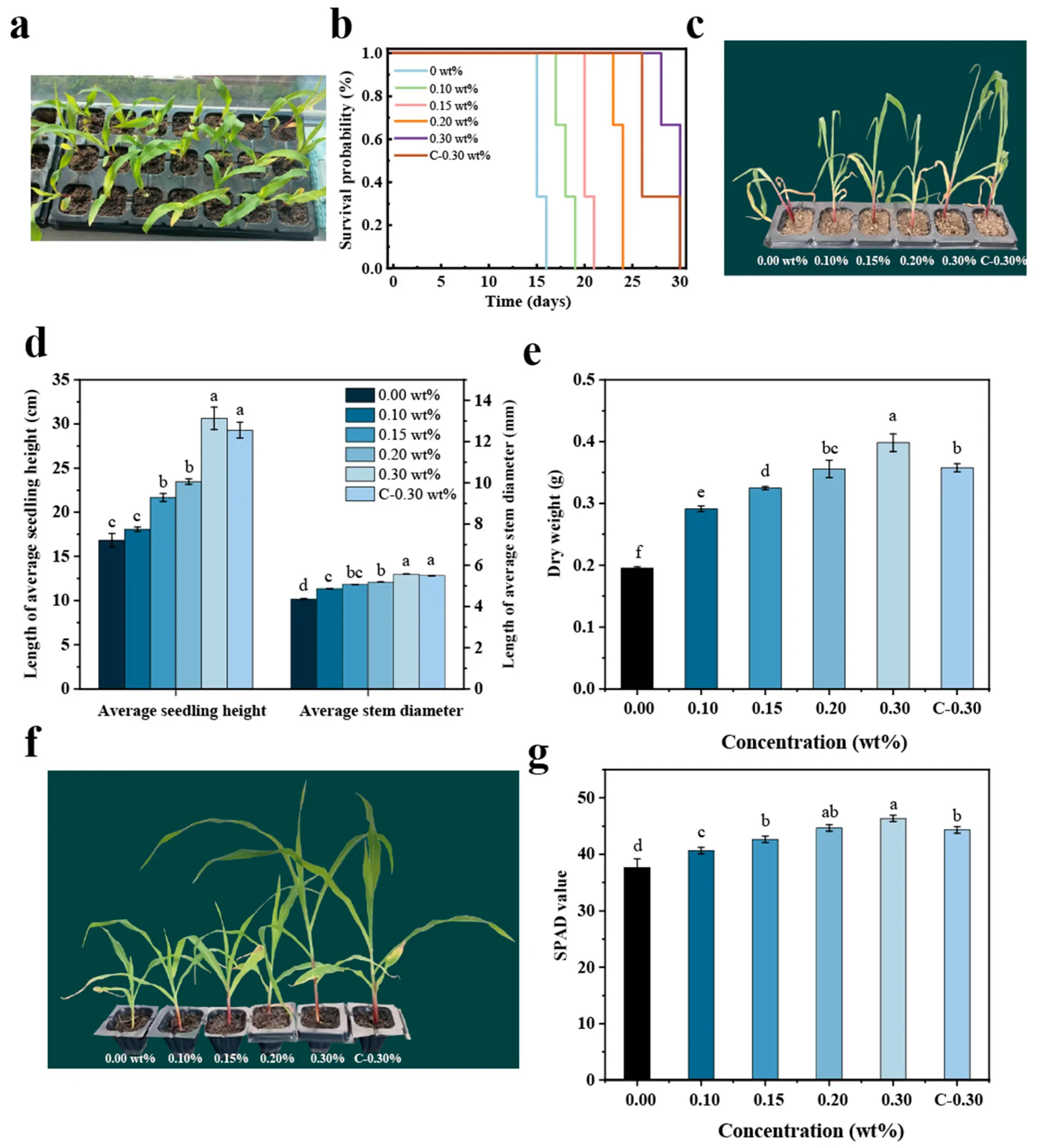
(**a**) Photographs of maize seedlings after 7 days of cultivation; (**b**) Kaplan–Meier survival curves of maize seedlings under drought stress; (**c**) Photograph of maize seedlings after 7 days without irrigation; (**d**) Average root lengths and average stem heights, (**e**) dry weight, (**f**) photograph, and (**g**) Soil–plant analysis development (SPAD) values of maize seedlings after 25 days of cultivation under L-CF/CMCAA-1ATP -treated groups (0, 0.10, 0.15, 0.20, and 0.30 wt%), and commercial hydrogel (C-0.30 wt%). Different lowercase letters (a–f) indicate significant differences (*p* < 0.05) among treatments for survival time and growth parameters. Error bars represent mean ± SD.

Based on the maize seedling cultivation experiment (Figure 10a), the agricultural application of the SAHs was evaluated. To investigate the influence of L-CF/CMCAA-1ATP dosage on drought resistance, maize seedlings were cultivated under drought stress conditions, where irrigation was terminated after 25 days of normal watering. Kaplan–Meier survival analysis showed that the median survival time increased from 15 days in the control group to 18, 20, 24, and 30 days in the 0.10, 0.15, 0.20, and 0.30 wt% L-CF/CMCAA-1ATP groups, respectively. Thus, the median survival time was prolonged by 3, 5, 9, and 15 days, respectively, compared with the control. In comparison, the 0.30 wt% commercial hydrogel group exhibited a median survival time of 26 days, representing an 11-day extension relative to the control (Figure 10b). Moreover, after 7 days without irrigation, seedlings grown in SAH-amended soils remained upright, whereas those in the blank group exhibited severe wilting and lodging (Figure 10c). These results indicate that L-CF/CMCAA-1ATP hydrogel effectively improved soil water retention and prolonged water availability for maize seedlings under drought conditions.

To further evaluate its effect on plant growth, maize seedlings were cultivated under normal irrigation conditions. As shown in Figure 10d–f, all growth parameters improved with increasing L-CF/CMCAA-1ATP dosage. Compared with the blank group, the application of 0.30 wt% L-CF/CMCAA-1ATP increased seedling height, stem diameter, and dry biomass by 13.8 cm, 0.12 cm, and 0.20 g, respectively (*p* < 0.0001). At the same dosage, the growth-promoting effect was more pronounced than that of the commercial hydrogel. After 25 days of cultivation, maize seedlings treated with L-CF/CMCAA-1ATP exhibited vigorous growth and favorable morphology (Figure 10f), indicating that the improved soil moisture environment effectively supported plant development.

The chlorophyll content of maize seedlings, expressed as SPAD values, is shown in Figure 10g. Compared with the blank group, the SPAD value increased by 21.56% in the 0.30 wt% L-CF/CMCAA-1ATP treatment (*p* < 0.0001), whereas the commercial hydrogel treatment showed an increase of 15.26% (*p* < 0.0001). The higher chlorophyll content suggests an improved photosynthetic potential and physiological status of maize seedlings, consistent with the observed increases in plant growth and biomass accumulation.

Overall, the enhanced drought tolerance and growth performance of maize seedlings demonstrate that L-CF/CMCAA-1ATP effectively improves the soil water environment and supports plant development. These results highlight the potential of this cellulose-based SAH as a soil amendment for agricultural water conservation and crop production.

### 2.9. Safety Assessment

**Figure 11:**
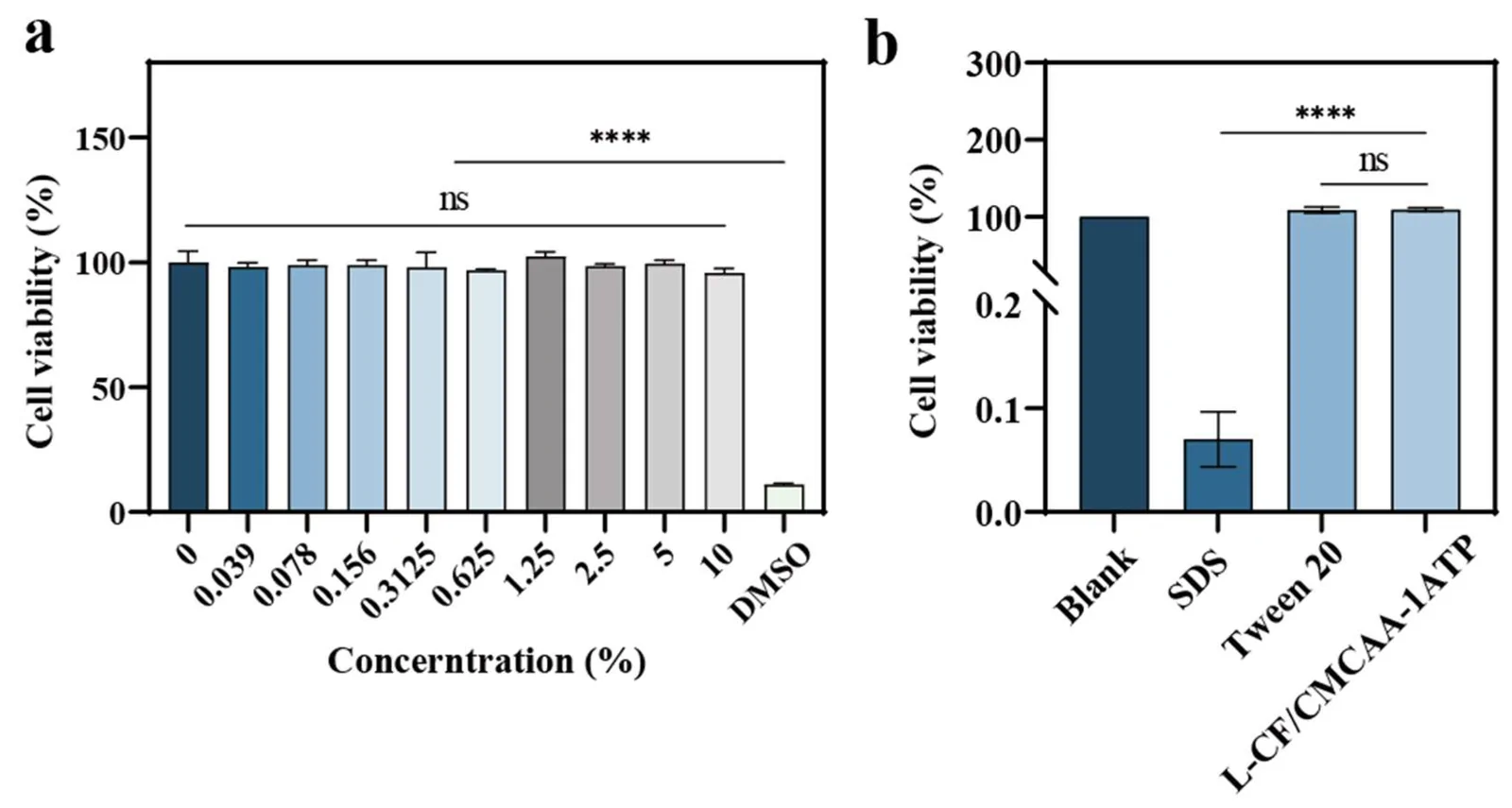
Cell viability of HaCaT cells (**a**) and SIRC cells (**b**) after treatment with L-CF/CMCAA-1ATP determined by the MTT assay. Error bars represent mean ± SD. ns, not significant (*p* ≥ 0.05). ****, *p* < 0.0001.

Although SAHs are mainly used as soil amendments, potential exposure during production, handling, and field application necessitates the evaluation of their cytotoxicity. The cytotoxicity of L-CF/CMCAA-1ATP hydrogel was assessed using MTT assays with HaCaT keratinocytes and SIRC corneal cells (Figure 11a,b). Across the tested concentration range (0.039–10%), cell viability remained above 95% in HaCaT cells, with no significant difference compared with the negative control (ns). In contrast, the positive control (10% dimethyl sulfoxide, DMSO) reduced cell viability to 10.99% (*p* < 0.0001), confirming the negligible cytotoxicity of L-CF/CMCAA-1ATP (Figure 11a). Ocular irritation was further evaluated using the SIRC assay (Figure 11b). While the positive control (0.5% sodium dodecyl sulfate, SDS) resulted in cell viability below 15%, L-CF/CMCAA-1ATP at 0.5% maintained a viability of 109.18%, comparable to that of Tween 20 (108.62%), indicating no detectable cytotoxicity under the tested conditions. Meanwhile, the favorable cytotoxicity results further support the low in vitro cytotoxicity of the hydrogel. Overall, these results indicate that L-CF/CMCAA-1ATP exhibits low cytotoxicity under the tested conditions, supporting its potential for agricultural applications.

## 3. Conclusions

Herein, a multifunctional cellulose-based SAH (L-CF/CMCAA-1ATP) was successfully synthesized via aqueous solution polymerization. The combined incorporation of carboxyl-functionalized cellulose fibers and attapulgite constructed a stable three-dimensional hydrogel network with excellent swelling, water retention, nutrient retention, and reusability. Soil application significantly improved water conservation, reduced nutrient leaching, and promoted maize growth, while DFT calculations revealed that H_2_PO_4_^−^ exhibited the strongest interaction with the hydrogel network, consistent with the experimental nutrient-retention behavior.

This work provides a waste valorization strategy for converting waste lotus stems into value-added carboxyl-functionalized cellulose reinforcements for superabsorbent hydrogels. The combined incorporation of L-CF and ATP simultaneously enhanced water retention, nutrient retention, and plant growth performance, demonstrating promising potential for cellulose-based SAHs in agricultural applications. While the current results demonstrate the promising potential of L-CF/CMCAA-1ATP for agricultural water and fertilizer management, field validation under diverse environmental conditions is essential to confirm its practical applicability and long-term performance. Future studies should also include quantitative soil-burial degradation experiments, with mass-loss measurements and representative observations over time, to determine the degradation rate and environmental persistence of the hydrogel under different soil conditions. Comprehensive environmental safety assessments, including soil ecotoxicity (e.g., earthworm acute toxicity and soil enzyme activity), aquatic toxicity, and long-term environmental fate monitoring, will be important for further evaluating its environmental safety for agricultural applications. Furthermore, future studies should also include more detailed physiological characterization of plant drought responses, such as photosynthetic activity, antioxidant enzyme activity, and water-status-related parameters, to further clarify the mechanisms underlying the observed drought tolerance.

## 4. Materials and Methods

### 4.1. Materials

Acrylic acid (AA, AR, 99%), carboxymethyl cellulose (CMC, USP grade), ammonium persulfate (APS, AR, 98%), sodium bisulfite (NaHSO_3_, AR), N,N′-methylenebisacrylamide (MBA, AR, 99%), choline chloride (AR, 98%), and anhydrous citric acid (AR, 99.5%) were purchased from Aladdin Biochemical Co., Ltd. (Shanghai, China). Sodium hydroxide (NaOH, AR, ≥96%), sodium bicarbonate (NaHCO_3_, AR, ≥99.7%), urea (AR, 99%) and potassium dihydrogen phosphate (AR, 99%) were supplied by Macklin Biochemical Co., Ltd. (Shanghai, China). Attapulgite (ATP) was provided by Titan Scientific Co., Ltd. (Shanghai, China). Human immortalized keratinocyte cells (HaCaT) and Statens Seruminstitut rabbit cornea cells (SIRC) were purchased from Haixing Biosciences Co., Ltd. (Suzhou, Jiangsu, China).

Analytical-grade reagents and ultrapure water were used throughout the study.

### 4.2. Preparation of Lotus Cellulose Fibers from Lotus Stem

A deep eutectic solvent (DES) was first prepared by mixing choline chloride, anhydrous citric acid, and deionized water at a mass ratio of 1:3:1. The mixture was heated to 80 ± 1 °C with continuous magnetic stirring until a transparent and homogeneous liquid was obtained. Fresh lotus stems were then cut into small pieces, pulverized, and sieved through a 50-mesh screen. The resulting powder was dispersed in the DES at a liquid-to-solid ratio of 20:1 (*w*/*w*) and reacted at 130 ± 3 °C under stirring (400 ± 5 rpm) for 3 h. Upon completion of the reaction, deionized water was introduced as an anti-solvent to regenerate the cellulose, followed by centrifugation at 2500× *g* for 40 min to recover the solid fraction. The collected solids were subsequently bleached using acidified sodium chlorite and repeatedly washed with deionized water until a neutral pH was reached. Finally, the purified cellulose was frozen at −80 °C and freeze-dried to obtain lotus cellulose fibers. The purified lotus cellulose fibers were then dispersed in deionized water to obtain a homogeneous 1 wt% suspension and subsequently spray-dried using an EYELA SD-1010 spray dryer. The resulting lotus cellulose powder (L-CF) was collected for further characterization and application studies.

### 4.3. Preparation of SAHs

To optimize the swelling performance of the SAHs, the effects of neutralization degree (ND), ATP content, and CMC content were systematically investigated, with all compositions expressed as wt% relative to AA. The ND was varied from 40% to 80% to determine the optimal neutralization condition. Subsequently, ATP content was adjusted from 1 to 7 wt% using the optimized CMCAA hydrogel formulation, followed by optimization of CMC content within the range of 3–15 wt%. Based on the optimized formulation, 2 wt% L-CF was incorporated to prepare the final L-CF/CMCAA-1ATP composite SAH.

For synthesis of the optimized SAHs, CMC and L-CF were first dispersed in deionized water under vigorous stirring (1200 rpm) to form a uniform suspension. NaOH was then added, followed by the slow addition of AA to control the neutralization process and associated heat release. Afterward, ATP powder was introduced and stirred to form a homogeneous dispersion. Finally, an aqueous initiator solution containing APS, MBA, NaHSO_3_, and NaHCO_3_ was added to initiate hydrogel network formation through polymerization. The reaction was conducted at 65 °C for 3 h to complete the polymerization. The crude product was repeatedly soaked in absolute ethanol (24 h each) to extract unreacted monomers, oligomers, and other soluble impurities, then dried in an oven at 65 °C and ground. Finally, the dried material was sieved to a particle size of 10–20 mesh for further characterization [10]. Upon hydration, the prepared dried material absorbed water and swelled to form SAHs. The specific amounts of each component used to prepare the optimized hydrogel are provided in Table S2.

For comparison, a pure sodium polyacrylate hydrogel sample (Pure PAA-Na), without CMC, L-CF and ATP, was prepared under the same conditions.

### 4.4. Characterization

The particle size distribution of L-CF was analyzed using a laser diffraction particle size analyzer (Mastersizer 1000, Malvern Panalytical, Malvern, UK). Prior to analysis, the samples were diluted 100-fold with ultrapure water and sonicated to achieve uniform dispersion and minimize multiple scattering effects.

^13^C solid-state nuclear magnetic resonance (ssNMR) spectroscopy was employed to examine the chemical structure of L-CF. Measurements were conducted on a Bruker Avance III spectrometer (Bruker BioSpin GmbH, Rheinstetten, Germany) equipped with a 4 mm MAS probe and operated at 9.4 T, corresponding to a ^13^C Larmor frequency of 100.61 MHz. The spectra were collected under magic-angle spinning at 14 kHz.

The carboxyl-group content of L-CF was determined by conductometric titration. Briefly, dried L-CF (0.10 g) was dispersed in 50 mL of deionized water and acidified with dilute HCl to convert the carboxylate groups into their protonated form. After equilibration under continuous stirring, the suspension was titrated with 0.01 mol/L NaOH solution while the conductivity was continuously recorded. The volume of NaOH corresponding to the neutralization of carboxyl groups was determined from the conductivity–titrant volume curve. The carboxyl-group content was calculated from the NaOH consumption and expressed as mmol/g of dry L-CF [60]. The degree of substitution was subsequently estimated based on the measured carboxyl-group content and the molar mass of the anhydroglucose unit.

The cellulose yield was determined gravimetrically from the dry mass of the recovered L-CF relative to the dry mass of the raw lotus stem powder. The cellulose content of L-CF was determined according to the NREL Laboratory Analytical Procedure. Briefly, dried samples were hydrolyzed with 72% H_2_SO_4_ at 30 °C for 60 min, followed by dilution to approximately 4% H_2_SO_4_ and further hydrolysis at 121 °C for 60 min. The released glucose was quantified by HPLC and converted to glucan content, which was used to determine the cellulose content on a dry-weight basis [61].

Fourier transform infrared (FTIR) spectroscopy was used to characterize the chemical structures of the raw materials (L-CF, CMC, and ATP) and the synthesized hydrogel (CMCAA and L-CF/CMCAA-1ATP). Prior to analysis, each sample was thoroughly blended with KBr, finely ground, and compressed into pellets. FTIR spectra were collected on a Nicolet iN10 spectrometer (Thermo Fisher Scientific, Waltham, MA, USA) over the wavenumber range of 4000–400 cm^−1^. The crystalline structures of L-CF, CMC, ATP, and the SAHs were examined using an X-ray diffractometer (D8 Advance, Bruker AXS GmbH, Karlsruhe, Germany) equipped with Cu Kα radiation (λ = 0.154 nm). Diffraction patterns were recorded over a 2θ range of 5–80° with a scanning rate of 0.5 s per step. Thermal stability was evaluated by thermogravimetric analysis (TGA 550, Discovery, TA Instruments, New Castle, DE, USA). The samples were heated from room temperature to 800 °C at a heating rate of 10 °C min^−1^ under a nitrogen atmosphere. The microstructures of the swollen hydrogels were observed using scanning electron microscopy (SEM, JSM-5600LV, Tokyo, Japan). Before SEM observation, the SAH samples were equilibrated in deionized water for 24 h, freeze-dried for 12 h, and sputter-coated with gold. Images were acquired at an accelerating voltage of 5 kV. Elemental distributions were further analyzed using an energy-dispersive X-ray spectroscopy (EDS) detector attached to the SEM at an accelerating voltage of 15 kV.

### 4.5. Performance of SAHs

#### 4.5.1. Equilibrium Swelling

Accurately weighed dried SAH samples (0.100 ± 0.001 g) were immersed in an appropriate medium (500 mL) at room temperature and allowed to swell freely until equilibrium was reached. The swollen samples were then separated from the free liquid using a 100-mesh nylon screen and allowed to drain until no continuous droplets were observed. Residual surface water was gently removed with filter paper without applying external pressure, and the swollen samples were immediately weighed. The equilibrium swelling ratio, Q_e_ (g/g), which represents the amount of water absorbed per unit dry mass of the hydrogel, was calculated according to formula (1)

(1) $$Q\left(g/g\right)=\frac{\left(M-M_{t}\right)}{M_{t}}$$

where M and M_t_ represent the weights of the swollen and dried samples, respectively [15]. All measurements were performed in triplicate.

Swelling behavior of the SAHs was also evaluated in different media, including tap water, rainwater, phosphate buffer solutions (pH 5 and pH 9), and a standard saline solution (0.9% NaCl), 0.01 mol/L CaCl_2_ solution, and 0.01 mol/L MgCl_2_ solution.

#### 4.5.2. Swelling Kinetics

The swelling kinetics of the SAHs in deionized water were evaluated as follows. Briefly, 0.10 g of dry samples was immersed in 500 mL of deionized water, and the swelling capacity was measured at predetermined time intervals (10, 20, 30, 50, 70, 120, 180, 240, and 300 min) [62]. The water absorption rate of the SAHs was calculated according to formula (1).

The swelling kinetics of the SAHs were further evaluated using the pseudo-second-order kinetic model and the Ritger–Peppas model, as expressed by formula (2) and formula (3), respectively:

(2) $$\frac{t}{Q_{t}}=\frac{1}{K_{2}Q_{e}^{2}}+\frac{t}{Q_{e}}$$

where Q_t_ and Q_e_ are the swelling capacities at time t and equilibrium, respectively, and K_2_ is the rate constant.

(3) $$\log\left(\frac{Q_{t}}{Q_{e}}\right)=\ln\left(k\right)+n\ln\left(t\right)$$

where k is the proportionality constant taking into account the properties of the hydrogel network and the penetrant, and n is the diffusion exponent used to characterize the water transport mechanism within the hydrogel matrix [17].

#### 4.5.3. Water Retention Capacity at Different Temperatures

The water retention performance and thermal stability of the SAHs were evaluated at 45 °C and 75 °C. In addition, the total water retention time at 75 °C was recorded.

Accurately weighed dry samples (0.1 g) were first immersed in deionized water to reach swelling equilibrium. The resulting fully swollen hydrogels were then placed in an oven maintained at the target temperatures (45 °C or 75 °C).

The weight of the hydrogel samples was recorded at specific time intervals (0, 30, 60, 90, 120, and 240 min). The water retention rate in deionized water (R_r_, %) was calculated using Formula (4).

(4) $$R_{r}(\%)=\frac{W_{t}-W_{d}}{W_{s}-W_{d}}\times 100$$

where R_r_ is the water retention rate; W_s_ is the weight of the fully swollen hydrogel; W_d_ is the weight of the initially dried sample; and W_t_ is the weight of the hydrogel sample at time point [63].

The hydrogel samples at 75 °C were kept in the oven and weighed periodically beyond 240 min until a constant weight was reached. The water retention time was defined as the elapsed time from the beginning of heating at 75 °C to the point at which the sample reached a constant weight, indicating complete loss of retained water.

#### 4.5.4. Reswelling Experiment

The reswelling behavior of the SAHs was evaluated over multiple swelling–drying cycles. Briefly, 0.10 g of dried sample was immersed in 500 mL of deionized water and allowed to swell at room temperature for 4 h to reach equilibrium. The fully swollen hydrogel was then dried in an oven at 105 °C until a constant weight was obtained. After drying, the sample was re-immersed in the same volume of deionized water to allow hydrogel reswelling. This hydrogel swelling–drying process was repeated for several cycles, and the swelling ratio was calculated for each cycle using the method described in Section 4.5.1 [64].

#### 4.5.5. Soil Water Retention Experiment

The soil used in the experiment was sandy soil with a bulk density of 1.45 g/cm^3^ and a pH of 7.1. Soil samples were air-dried and passed through a 20-mesh nylon screen. The samples were mixed thoroughly with the dried soil at 0, 0.10, 0.15, 0.20 and 0.30 wt% and then blended with soil (200 g) prior to filling the PVC pipe (4.5 cm diameter) of the test apparatus. Additionally, a commercial hydrogel (sodium polyacrylate hydrogel) was mixed with the soil at 0.30 wt% (C-0.30 wt%) as a positive control.

Tap water was slowly added from the top until water began to drain from the bottom. The water holding capacity was recorded at the point when no further drainage occurred. The water retention capacity was then monitored by weighing the columns over 21 days at ambient temperature (22 ± 3 °C).

The water holding capacity (W_h_, %), which refers to the amount of water retained by the soil–hydrogel system after saturation and complete drainage, and water retention capacity (W_r_, %) of the soil, which describes the fraction of water remaining in the system over time, were calculated from formula (5) [65] and formula (6) [47].

(5) $$W_{h}(\%)=\frac{W_{1}-W_{0}}{200}\times 100$$

where W_0_ and W_1_ are the weights of the dried and saturated samples/soil samples respectively.

(6) $$W_{r}\left(\%\right)=\frac{W_{i}-W_{0}}{W_{1}-W_{0}}\times 100$$

where W_i_ is the weight of the SAHs/soil at each time point.

#### 4.5.6. Fertilizer Retention Experiment

The fertilizer-retention performance of the SAHs was evaluated by monitoring nitrogen, phosphorus, and potassium leaching. Briefly, 125 g of oven-dried soil, sieved through a 10-mesh sieve, was packed at the bottom of each PVC column (5 cm inner diameter × 40 cm height) as a buffer layer. Urea and potassium dihydrogen phosphate (KH_2_PO_4_) were used as nutrient sources for N, P, and K. Specifically, 110 mg of urea and 200 mg of KH_2_PO_4_ were dissolved in deionized water to prepare the nutrient solution. Hydrogels at different application rates (0, 0.10, 0.15, 0.20, and 0.30 wt%) were allowed to swell to equilibrium in the nutrient solution to form nutrient-loaded hydrogels. The control group (0 wt%) received the same amounts of urea and KH_2_PO_4_ but without hydrogel. The nutrient-loaded hydrogels were then mixed uniformly with another 125 g of soil and loaded into the column above the buffer layer. Each treatment was performed in triplicate. The columns were sealed and equilibrated for 48 h before leaching. For each leaching event, 100 mL of deionized water was added dropwise from the top of the column at a rate of approximately one drop per minute. Leaching was performed at 5-day intervals for a total of five cycles. The leachate collected from each cycle was diluted with deionized water to a final volume of 250 mL prior to nitrogen, phosphorus, and potassium analysis.

Total nitrogen was determined after alkaline potassium persulfate digestion using a UV–Vis spectrophotometer (UV-8100B, LabTech Holdings, Inc., Beijing, China). Phosphorus and potassium concentrations were measured using inductively coupled plasma optical emission spectroscopy (ICP-OES, Avio 8300, PerkinElmer, Waltham, MA, USA).

#### 4.5.7. Maize Seedlings Planting Experiment

The maize seedling experiments were conducted at the Harbin Institute of Technology (Shenzhen) under controlled conditions (26 ± 2 °C and 65 ± 5% relative humidity) from August 2025 to September 2025. The breeding substrate was a mixture of peat, coconut bran, wood pellet and charred rice husk in a specific proportion. The basic properties of the substrate were pH 6.5, organic matter content 68.4%, total nitrogen 0.62%, available phosphorus 48.6 mg/kg, and available potassium 356.2 mg/kg. No additional fertilizer was applied during the cultivation period. Each treatment consisted of three independent pots, which were considered biological replicates (n = 3). Maize seeds (cv. Zhengdan 985) were randomly selected and pre-soaked in a 40 ± 2 °C water bath using the moist paper towel method to initiate germination. Successfully sprouted seeds were sown individually at a depth of 2 cm in pots containing 150 cm^3^ of a substrate mixture. The cultivation substrate consisted of a formulated blend of peat, coir (coconut bran), wood pellets, and carbonized rice husks in predetermined proportions. For the first four days, each pot was irrigated with 20 mL of deionized water daily. Starting from the fourth day post-sowing, an irrigation regimen of 20 mL every three days was implemented. The seedlings were cultivated for a total duration of 25 days prior to harvest and subsequent analysis.

Two parallel maize seedling experiments were conducted to evaluate drought tolerance and, separately, growth performance under normal water supply. In both experiments, L-CF/CMCAA-1ATP was incorporated into the cultivation substrate at 0, 0.10, 0.15, 0.20, and 0.30 wt%, and a commercial hydrogel treatment (C-0.30 wt%) was included for comparison. Each treatment consisted of three independent pots.

For the drought stress group, regular irrigation was applied for 25 days to ensure seedling establishment, after which irrigation was completely withheld to simulate drought conditions. The growth status of maize seedlings was continuously monitored, and the survival time was recorded until plant death. For the normal water supply group, regular irrigation was maintained throughout the entire growth period.

At harvest, plant height and stem diameter were measured. The seedlings were then rinsed with deionized water to remove substrate, blotted dry, and oven-dried at 65 °C to constant weight for biomass determination [66]. The relative chlorophyll content of maize seedling leaves was measured using a soil–plant analysis development (SPAD) chlorophyll meter (SPAD-502 PLUS, Konica Minolta, Tokyo, Japan).

#### 4.5.8. Determination of Gel Fraction and Sol Fraction

The gel fraction and sol fraction of the hydrogels were determined by an extraction–drying method. Briefly, the dried hydrogel samples were weighed (W_0_) and immersed in excess deionized water for 48 h to remove soluble and unreacted components, with the water replaced periodically. The remaining insoluble hydrogel was collected and dried to a constant weight (W_d_). The gel fraction and sol fraction were calculated as follows:

(7) $$\mathrm{Gel}\;\mathrm{fraction}\;(\%)\;=\;\frac{W_{d}}{W_{0}}\times 100$$

(8) $$\mathrm{Sol}\;\mathrm{fraction}\;(\%)=\frac{W_{0}-W_{d}}{W_{0}}\times 100$$

### 4.6. DFT Calculations

All calculations were performed using Gaussian 16 A.03 software [67]. The B3LYP functional [68], combined with Grimme’s DFT-D3(BJ) [69,70] dispersion correction, was employed for all calculations. Geometry optimizations were carried out using the 6–31G* basis set [71,72] for all atoms. The polarizable continuum model with water as the solvent was employed during geometry optimization to account for aqueous solvation effects. Considering the heterogeneous and polymeric nature of the hydrogel, representative finite molecular fragments were employed to describe the local chemical environments of its major components rather than the complete crosslinked hydrogel network. Following the representative-fragment strategy commonly used for CMC-based systems [73], CMC was modeled using a carboxymethyl-substituted glucose unit representing the local structure of the cellulose backbone and its carboxymethyl functionality. L-CF was represented by a two-glucose-unit cellulose fragment bearing a citric-acid-derived substituent at a hydroxyl site to reproduce its local carboxyl-functionalized environment. The ATP model was constructed by extracting a representative finite cluster from the crystal structure of palygorskite. The resulting ATP cluster was treated as a neutral closed-shell system with a total charge of 0 and a spin multiplicity of 1. For the ATP cluster, an additional calculation was performed at the B3LYP-D3(BJ)/def2-TZVPP level using the SMD implicit water-solvation model. The ionization states of the functional groups were assigned according to the chemical states represented in the corresponding molecular models. Carboxyl groups were modeled in their protonated (–COOH) or deprotonated (–COO^−^) forms, as appropriate, whereas hydroxyl groups were retained in their neutral protonated form. Counterions were included where required to maintain chemically reasonable charge states. The total charge and spin multiplicity of each molecular model were specified prior to calculation. Nutrient species were represented separately as urea, H_2_PO_4_^−^, and K^+^. Wavefunction analyses were performed using the Multiwfn 3.8(dev) program [74,75]. The electrostatic potential (ESP) extrema projected onto the van der Waals surface were obtained through quantitative analysis of the electrostatic potential surface [76]. The weak noncovalent interactions were analyzed using the independent gradient model based on the Hirshfeld partition (IGMH) [77] and atoms-in-molecules (AIM) methods implemented in Multiwfn, and the resulting interaction isosurfaces were rendered with VMD [78]. Because these calculations employed representative finite molecular fragments and implicit aqueous solvation models, the calculated interactions were interpreted as complementary molecular-level evidence for possible hydrogel–nutrient interactions rather than as a complete representation of the heterogeneous hydrogel or soil environment.

### 4.7. Cytotoxicity Assay of L-Cf/cmcaa-1atp

Cytotoxicity of L-CF/CMCAA-1ATP hydrogel was evaluated using HaCaT and SIRC cell lines cultured in DMEM supplemented with 10% FBS and 1% antibiotics. Prior to cytotoxicity testing, the synthesized hydrogel was thoroughly washed with deionized water to reduce residual unreacted monomers and other water-soluble compounds. Cells were seeded in 96-well plates at 1 × 10^4^ cells/well and incubated overnight at 37 °C under 5% CO_2_. HaCaT cells were exposed to a range of L-CF/CMCAA-1ATP concentrations for 24 h, with untreated cells as negative control and 10% DMSO as positive control. SIRC cells were treated with 0.5% L-CF/CMCAA-1ATP for 24 h, using PBS as a negative control, Tween 20 (5%) as a mild irritant reference, and SDS (0.5%) as a positive control. After incubation, cells were washed, treated with MTT solution (10% *v*/*v*), incubated for 4 h, and then solubilized in DMSO. Absorbance at 490 nm was measured using a microplate reader (EPOCH2, BioTek Instruments, Inc., Winooski, VT, USA), and cell viability was calculated according to formula (9) [79]:

(9) $$\mathrm{Cell}\;\mathrm{viability}\;\left(\%\right)=\frac{A_{\mathrm{test}}-A_{\mathrm{blank}}}{A_{\mathrm{control}}-A_{\mathrm{blank}}}\times 100\%$$

where A_test_, A_blank_ and A_control_ are the mean absorbances of the sample-treated cells, untreated cells, and cell-free blank, respectively.

### 4.8. Statistical Analysis

All statistical analyses were performed using GraphPad Prism (version 9.5, GraphPad Software, San Diego, CA, USA) and OriginPro (version 2021, OriginLab Corporation, Northampton, MA, USA). All experimental measurements were performed in triplicate using independently measured samples, and the results are presented as mean ± SD. Each treatment consisted of three independent pots, with each pot considered an independent biological replicate (n = 3). Unpaired Student’s t-test was used for two-group comparison and one-way analysis of variance for multigroup analysis. Survival under drought stress was analyzed using the Kaplan–Meier method, with seedling death defined as the event and differences among groups evaluated using the log-rank (Mantel–Cox) test. Asterisks represent statistically significant differences (* *p* < 0.05; ** *p* < 0.01; *** *p* < 0.001; **** *p* < 0.0001; ns, not significant, *p* ≥ 0.05). In some figures, different letters (a, b, c, …) are also used to denote significant differences among multiple groups.

## Figures and Tables

**Table 1:** The swelling kinetics parameters of SAHs in deionized water at different times.

Sample	Swelling Kinetic Equation				n		k	
	10–50 min	R^2^	50–300 min	R^2^	10–50 min	50–300 min	10–50 min	50–300 min
L-CF/CMCAA-1ATP	Y = 0.15507x − 0.29531	0.9305	Y = 0.04427x − 0.10574	0.8439	0.1551	0.0443	5.08 × 10^−1^	7.85 × 10^−1^
CMCAA-1ATP	Y = 0.14232x − 0.31170	0.9197	Y = 0.0803x − 0.19176	0.9248	0.1423	0.0803	4.877 × 10^−1^	6.440 × 10^−1^
CMCAA	Y = 0.17583x − 0.33357	0.9810	Y = 0.04159x − 0.10365	0.8543	0.1758	0.0416	4.638 × 10^−1^	7.890 × 10^−1^
Commercial	Y = 0.23352x − 0.45006	0.8997	Y = 0.09094x − 0.21711	0.9309	0.2335	0.0909	3.548 × 10^−1^	6.080 × 10^−1^
Pure PAA-Na	Y = 0.74619x − 1.39637	0.9925	Y = 0.18273x − 0.428	0.7845	0.7462	0.1827	0.401 × 10^−1^	3.743 × 10^−1^

## Data Availability

The original contributions presented in this study are included in the article/Supplementary Material. Further inquiries can be directed to the corresponding author.

## Supplementary Materials

The following supporting information can be downloaded at: https://www.mdpi.com/article/10.3390/gels12090854/s1, Figure S1: EDS elemental analysis and sum spectrum of L-CF/CMCAA-1ATP; Table S1: Kinetic parameters for pseudo-second-order models; Table S2: Complete formulation of the optimized L-CF/CMCAA-1ATP hydrogel; Table S3: Initial amounts of N, P, and K applied and cumulative amounts recovered in the leachate during five leaching times; Table S4: Abbreviations.
