# Supplementary material for: Carboxyl-Functionalized Lotus Cellulose Fiber-Based Superabsorbent Hydrogel for Efficient Water and Fertilizer Management
Source: Gels. 2026 Sep 20;12(9):854. doi: 10.3390/gels12090854 (PMC13606061; doi:10.3390/gels12090854)
Supplement: Supplementary file 1 [file gels-12-00854-s001.zip › gels-4544880-Supplementary material.pdf]

Research paper

Carboxyl-Functionalized Lotus Cellulose Fiber-Based Superabsorbent Hydrogel for Efficient Water and Fertilizer Management

Jiawen Chen[a,b,c,1], Guohang Ma[e,1], Keting Chen[d], Tiansheng Wang[a,b,c], Erlei Zhang[b], Jianmin Li[d], Bao Han[d], Wenfeng Lu[d], Jichuan Zhang[a,b,c,d], Jiaheng Zhang[a,b,c,d,*]

[a] Sauvage Laboratory for Smart Materials, Harbin Institute of Technology (Shenzhen), 518055, China;

[b] School of Materials Science and Engineering, Harbin Institute of Technology (Shenzhen), 518055, China;

[c] State Key Laboratory of Advanced Welding and Joining and Research Centre of Printed Flexible Electronics, School of Materials Science and Engineering, Harbin Institute of Technology (Shenzhen), 518055, China;

[d] Shenzhen Shinehigh Innovation Technology Co., Ltd, Shenzhen, 518055, China

[e] School of Life Sciences, Jilin University (Changchun), 130000, China

[*]Corresponding author at: Sauvage Laboratory for Smart Materials, Harbin Institute of Technology, Shenzhen, 518055, China. *E-mail address*: zhangjiaheng@hit.edu.cn

Part I: Supplementary Figures

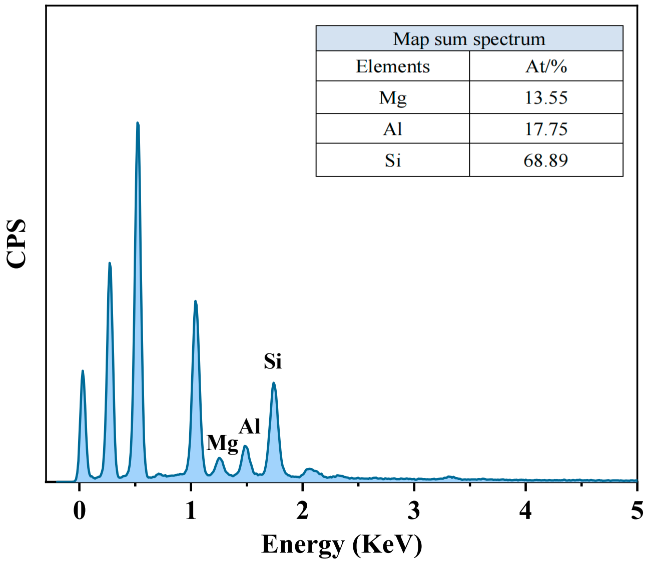

Figure S1. EDS elemental analysis and sum spectrum of L-CF/CMCAA-1ATP.

Part II: Supplementary Tables

Table S1. Kinetic parameters for pseudo-second-order models.

Samples	Pseudo-second-order model			
	Q_e, cal. (g/g)	$k_2 \times 10^{-4}$ (g/g·min)	K_{is} (g/g·min)	R^2
L-CF/CMCAA-1ATP	2009	1.06	427.35	0.9997
CMCAA-1ATP	1409	0.88	175.13	0.9999
CMCAA	885	2.16	168.92	0.9998
Commercial	500	2.27	56.82	0.9998
Pure PAA-Na	256	1.20	7.83	0.9946

Table S2. Complete formulation of the optimized L-CF/CMCAA-1ATP hydrogel.

Component	Actual amount (g)
Acrylic acid (AA)	20.00
Sodium hydroxide (for ND=60%)	6.66
Carboxymethyl cellulose (CMC)	1.00
Attapulgite (ATP)	0.20
L-CF	1.00
APS	0.20
MBA	0.02
$NaHSO_3$	0.10
$NaHCO_3$	0.20
Deionized water	20.00

Table S3. Initial amounts of N, P, and K applied and cumulative amounts recovered in the leachate during five leaching times.

Nutrient	Treatment	Initial amount applied (mg)	Leaching 1 (mg)	Leaching 2 (mg)	Leaching 3 (mg)	Leaching 4 (mg)	Leaching 5 (mg)
N	0 wt%	51.3 ± 0.5	8.10 ± 0.30	21.89 ± 0.64	33.60 ± 0.46	37.11 ± 0.46	38.63 ± 0.38
N	0.10 wt%	51.3 ± 1.0	8.38 ± 0.08	18.22 ± 0.49	30.30 ± 0.23	33.04 ± 0.68	35.12 ± 0.68
N	0.15 wt%	51.3 ± 0.8	7.71 ± 0.30	18.73 ± 0.35	29.68 ± 0.82	31.70 ± 0.36	32.79 ± 0.86
N	0.20 wt%	51.3 ± 0.6	6.56 ± 0.41	15.32 ± 0.72	26.01 ± 0.67	28.44 ± 0.53	29.95 ± 0.42
N	0.30 wt%	51.3 ± 0.3	5.26 ± 0.21	13.82 ± 0.05	23.33 ± 0.17	25.47 ± 0.37	27.49 ± 1.03
P	0 wt%	45.5 ± 1.2	4.04 ± 0.09	7.54 ± 0.08	10.88 ± 0.41	12.50 ± 0.42	14.28 ± 0.33
P	0.10 wt%	45.5 ± 0.5	3.60 ± 0.10	7.38 ± 0.37	10.46 ± 0.36	12.31 ± 0.57	13.53 ± 0.49
P	0.15 wt%	45.5 ± 0.9	2.71 ± 0.24	5.14 ± 0.12	8.37 ± 0.11	9.76 ± 0.14	12.53 ± 0.71
P	0.20 wt%	45.5 ± 1.0	1.52 ± 0.25	4.37 ± 0.21	7.27 ± 0.27	9.00 ± 0.22	10.78 ± 0.23
P	0.30 wt%	45.5 ± 0.6	0.87 ± 0.16	2.97 ± 0.65	6.13 ± 0.37	8.26 ± 0.10	10.20 ± 0.62
K	0 wt%	58.0 ± 1.3	12.08 ± 0.56	32.09 ± 0.69	41.11 ± 1.02	45.40 ± 0.44	47.94 ± 0.38
K	0.10 wt%	58.0 ± 0.7	9.61 ± 0.62	24.02 ± 0.44	31.92 ± 0.59	37.50 ± 0.65	39.73 ± 0.27
K	0.15 wt%	58.0 ± 2.4	6.68 ± 0.21	17.54 ± 0.45	24.50 ± 0.26	35.52 ± 0.51	36.85 ± 0.14
K	0.20 wt%	58.0 ± 1.8	6.76 ± 0.44	11.85 ± 1.30	18.59 ± 0.33	27.74 ± 0.38	29.65 ± 1.09
K	0.30 wt%	58.0 ± 0.5	2.96 ± 0.09	11.02 ± 0.54	16.60 ± 0.61	25.61 ± 0.48	27.69 ± 0.52

Table S4. Abbreviations

Abbreviation	Full Name
AA	acrylic acid
AIM	atoms-in-molecules
APS	ammonium persulfate
ATP	attapulgite
CMC	carboxymethyl cellulose
CMCAA	carboxymethyl cellulose/acrylic acid hydrogel
DES	deep eutectic solvent
DFT	density functional theory
DMSO	dimethyl sulfoxide
EDS	energy-dispersive X-ray spectroscopy
ESP	electrostatic potential
FTIR	Fourier transform infrared spectroscopy
HaCaT	human immortalized keratinocyte cells
ICP-OES	inductively coupled plasma optical emission spectroscopy
IGMH	independent gradient model based on Hirshfeld partition
L-CF	lotus cellulose fibers
MBA	N,N'-methylenebisacrylamide
ND	neutralization degree
ρBCP	bond critical point
SAHs	superabsorbent hydrogels
SDS	sodium dodecyl sulfate
SEM	scanning electron microscopy
SIRC	statens seruminstitut rabbit cornea cells
SPAD	soil plant analysis development
TGA	thermogravimetric analysis
XRD	x-ray diffraction

ssNMR	^{13}C solid-state nuclear magnetic resonance
